# NKG2C and NKG2A coexpression defines a highly functional antiviral NK population in spontaneous HIV control

**DOI:** 10.1172/jci.insight.182660

**Published:** 2024-09-17

**Authors:** Nerea Sánchez-Gaona, Ana Gallego-Cortés, Antonio Astorga-Gamaza, Norma Rallón, José Miguel Benito, Ezequiel Ruiz-Mateos, Adrian Curran, Joaquin Burgos, Jordi Navarro, Paula Suanzes, Vicenç Falcó, Meritxell Genescà, Maria J. Buzon

**Affiliations:** 1 Infectious Diseases Department, Hospital Universitari Vall d’Hebron, Institut de Recerca (VHIR), Universitat Autònoma de Barcelona, Barcelona, Spain.; 2 HIV and Viral Hepatitis Research Laboratory, Instituto de Investigación Sanitaria-Fundación Jiménez Díaz, Universidad Autónoma de Madrid (IIS-FJD, UAM), Madrid, Spain.; 3 Hospital Universitario Rey Juan Carlos, Móstoles, Spain.; 4 Institute of Biomedicine of Seville (IBiS), Virgen del Rocío University Hospital, Consejo Superior de Investigaciones Científicas (CSIC), University of Seville, Clinical Unit of Infectious Diseases, Microbiology and Parasitology, Seville, Spain.

**Keywords:** AIDS/HIV, Innate immunity

## Abstract

Elite controllers (ECs), a unique group of people with HIV (PWH), exhibit remarkable control of viral replication in the absence of antiretroviral therapy. In this study, we comprehensively characterized the NK cell repertoire in ECs after long-term viral control. Phenotypic profiling of NK cells revealed profound differences compared with other PWH, but marked similarities to uninfected individuals, with a distinctive prevalence of NKG2C^+^CD57^+^ memory-like NK cells. Functional analyses indicated that ECs had limited production of functional molecules upon NK stimulation and consequently reduced natural cytotoxicity against non-HIV target cells. Importantly, ECs showed an exceptional ability to kill primary HIV-infected cells by the antibody-dependent cell cytotoxicity adaptive mechanism, which was achieved by a specific memory-like NK population expressing CD16, NKG2A, NKG2C, CD57, and CXCR3. In-depth single-cell RNA-seq unveiled a unique transcriptional signature in these NK cells linked to increased cell metabolism, migration, chemotaxis, effector functions, cytokine secretion, and antiviral response. Our findings underscore a pivotal role of NK cells in the immune control of HIV and identify specific NK cells as emerging targets for immunotherapies.

## Abstract 2

People with spontaneous control of HIV have a unique immune response, characterized by a specific subset of natural killer (NK) cells that effectively kill HIV-infected cells through antibody-dependent cell cytotoxicity.

## Graphical abstract

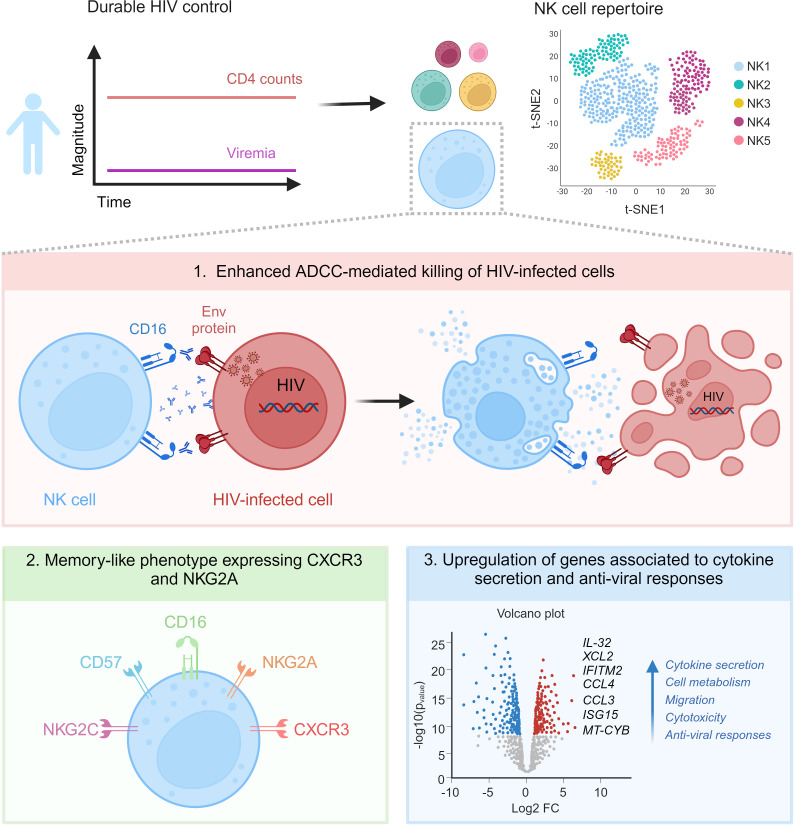

## Introduction

Elite controllers (ECs) are extraordinary people with HIV (PWH) who maintain undetectable HIV viremia in the absence of antiretroviral therapy (ART) (1, 2). Although ECs cannot eliminate the HIV infection, their immune system presents a remarkable ability to control viral replication, representing a model for a functional cure (3–6). Fundamental to this exceptional control, there are different genetic, immunological, and virological factors that vary among EC cohorts and contribute to their ability to maintain viral suppression (7, 8). The establishment and duration of viral control after acute infection is also not uniform across all ECs and depends on both viral and host factors. This suggests that effective host mechanisms from the beginning of infection are essential to achieve rapid suppression of viral replication (9–11). Only a small proportion (around 0.15%) can maintain replication control for long periods (longer than 10 years) (12, 13).

Genetic polymorphisms in the human HLA class I locus, particularly the overrepresentation of alleles including HLA-B*57, HLA-B*27, HLA-B*13, and HLA-B*58-01, are strongly associated with HIV control (14, 15). Some studies have demonstrated the association of these protective MHC alleles with a robust cytotoxic CD8^+^ T cell response in ECs (16, 17). Nonetheless, a considerable proportion of ECs that lack protective MHC alleles may control HIV infection through non-CTL mechanisms, pointing out that MHC alleles are neither necessary nor sufficient for HIV control (18). While studies on ECs have intensively focused on adaptive immunity due to the association with MHC-I alleles (19, 20), it is possible that innate immune responses are also important in HIV control (21, 22).

Natural killer (NK) cells are critical effector cells from the innate immunity that play an important role in HIV infection since they recognize and respond to HIV-infected cells from the earliest infection stages (23). The NK cell activation state is contingent on the signaling received through a broad set of cell surface receptors. When these receptors bind to their cognate ligands, they promote either activating or inhibitory signals. Therefore, the balance between these opposing signals dictates the functional status of NK cells (24). Activating receptors such as NKG2C, NKp30, and NKG2D facilitate target cell recognition and cytotoxicity, while inhibitory receptors such as KLRG1, NKG2A, and killer immunoglobulin-like receptors (KIRs) help fine-tune NK cell responses by counteracting excessive activation (25). This balance serves to educate NK cells, promoting tolerance to healthy cells while priming them to respond to infected cells with reduced MHC-I antigen expression levels (26). In HIV infection, certain genetic combinations, such as KIR3DS1 with its ligand HLA-Bw4-08I, and KIR3DL1 with HLA-B*57, have been associated with lower viral loads and reduced risk of progression to AIDS, emphasizing the importance of NK cell genetics in HIV control (27, 28). Importantly, during viral infections such as with human cytomegalovirus (CMV), the virus can imprint the NK cell receptor repertoire, leading to the expansion of diverse, long-lived, and highly functional NK cells (29). These expanded NK cells, characterized by the expression of NKG2C and CD57, have the potential to mediate immune memory (30).

In addition, NK cells mediate antibody-dependent cellular cytotoxicity (ADCC), an adaptive immune mechanism implicated in protection against HIV (31). ADCC is triggered when NK cells, via CD16, recognize the fragment crystallizable (Fc) region of IgG antibodies bound to aberrant targets. Antibodies form a bridge between NK and target cells, promoting CD16 cross-linking, which triggers NK cell activation and degranulation, leading to the cytolysis of target cells. In this regard, several studies have shown that PWH present an altered NK cell phenotype and decreased cytotoxic activity compared with healthy individuals (32–34). Other studies have reported elevated levels of anti-Env/gp120 antibodies in ECs compared with other groups of PWH (31). However, the specific contribution of NK cells in HIV control via the ADCC mechanism remains unclear in these individuals. Taken together, these studies provide strong evidence that NK cells influence disease progression, and therefore, represent a target for further exploration in the context of HIV control.

Here, to identify NK cell factors associated with HIV control, we investigated the phenotypic, functional, and transcriptional profiles of NK cells from 4 cohorts of PWH: ART-suppressed (ART), ART-naive viremic (VIR), ECs with durable HIV control (DC), or immunological aborted HIV control (AC), compared with uninfected individuals (HD). In-depth characterization indicated that DC individuals present a unique NK cell repertoire associated with a robust ADCC response against HIV-infected cells. Our work shows important aspects of the NK cell phenotype and function in ECs that may contribute to the long-term HIV control, and which might have important implications for the development of NK-targeted anti-HIV immunotherapies.

## Results

### Cohort characteristics.

PBMCs from 34 ECs were obtained from the Spanish AIDS Research Network (RIS) cohort of HIV Controllers Study Group (ECRIS). Within this cohort, 21 participants demonstrated DC over HIV infection, maintaining viral loads below 40 HIV-1 RNA copies/mL and stable CD4^+^ T cell counts for a period ranging from 5 to 20 years (median 7.25 years). In contrast, 13 individuals experienced a loss of immunological control (AC), as evidenced by a progressive and statistically significant decline in CD4^+^ T cell count over the follow-up period (*P* < 0.05, determined by simple linear regression). The longitudinal profiles of CD4^+^ T cell counts and HIV-1 RNA levels for EC participants are depicted in Supplemental Figure 1; supplemental material available online with this article; https://doi.org/10.1172/jci.insight.182660DS1 Two additional cohorts were included in the study: samples from 2 to 4 years of ART (*n* = 24), and samples from VIR participants (*n* = 18). Samples from HD (*n* = 24) served as controls. The clinical characteristics of HIV^+^ individuals included in the study are summarized in Supplemental Table 1.

No significant differences in sex distribution were observed among the study groups (Supplemental Table 1). However, the median ages of DC (55 years) and AC (58 years) individuals were higher than that of ART (44 years) and VIR (39 years) groups (*P* < 0.05, Kruskal-Wallis test followed by Dunn’s multiple-comparisons test). These findings are consistent with the increased time since HIV diagnosis observed in the DC and AC groups compared with VIR (*P*_DC_ = 0.07, *P*_AC_ < 0.001, Kruskal-Wallis test followed by Dunn’s multiple-comparisons test). Furthermore, significant differences in transmission routes were observed among all groups (*P* < 0.0001, Fisher’s exact test). CD4^+^ T cell counts were also higher in DC, AC, and ART groups compared with VIR (*P* < 0.05, Kruskal-Wallis test followed by Dunn’s multiple-comparisons test). While DC, AC, and ART groups exhibited effective viral suppression (HIV-1 RNA < 40 copies/mL), VIR participants demonstrated viral loads ranging between 60,000 and 800,000 copies/mL (*P* < 0.0001, Kruskal-Wallis test followed by Dunn’s multiple-comparisons test). Despite having HLA typing data available for only 22 out of 34 participants within the EC group, protective HLA-B alleles (HLA-B57, B52, B27, and B14) (35) were found at comparable frequencies in both AC and DC participants (Supplemental Table 1). These findings suggest similarities in clinical, genetic, and virologic parameters within HIV^+^ individuals with spontaneous HIV control, while confirming the anticipated differences between these groups and viremic individuals.

### NK cell phenotypes with memory-like attributes distinguish individuals with DC.

**Figure 1:**
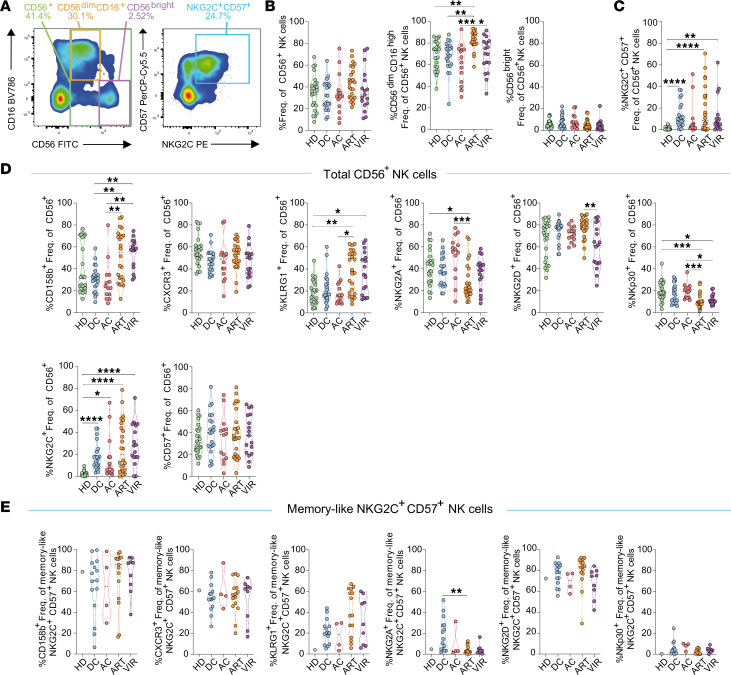
Phenotypic characterization of NK cells in ECs. The expression of different NK markers was quantified by flow cytometry in different study groups: healthy donors (HD, *n* = 25), ECs with durable HIV control (DC, *n* = 21), ECs with aborted immunological control (AC, *n* = 13), PWH ART-treated individuals (ART, *n* = 24), and viremic PWH (VIR, *n* = 18). (**A**) Representative flow cytometry plots depicting the NK cell subset gating strategy from CD3^–^ cells based on CD56 and CD16 expression (left: CD56^+^ total, CD56^dim^CD16^hi^, and CD56^bright^) and NKG2C and CD57 expression (right: NKG2C^+^CD57^+^ memory-like NK cells). (**B**) Violin plots depicting the frequency of different NK cell populations identified (left to right: CD56^+^ total, CD56^dim^CD16^high^, and CD56^bright^). (**C**) Violin plots depicting the frequency of memory-like NKG2C^+^CD57^+^ NK cells. (**D**) Violin plots depicting the frequency of different NK cell markers in CD56^+^ total NK cells by study group (left to right: CD158b, CXCR3, KLRG1, NKG2A, NKG2D, NKp30, NKG2C, and CD57). (**E**) Violin plots depicting the frequency of distinct NK cell receptors in expanded memory-like NKG2C^+^CD57^+^ NK cells (frequency >5% and counts >25; left to right: CD158b, CXCR3, KLRG1, NKG2A, NKG2D, and NKp30). Median with range is represented. Statistical comparisons were performed using Kruskal-Wallis 1-way ANOVA followed by Dunn’s multiple-comparison test. *P < 0.05; **P < 0<01; ***P < 0.001; ****P < 0.0001.

We first studied the overall frequencies of NK cells, the prevalence of the primary NK cell subsets based on CD56 and CD16 expression, and the percentage of memory-like NK cells characterized by the presence of NKG2C and CD57 markers across all study groups (36–38). An example of the gating strategy and a representative flow cytometry plot delineating the distinct NK cell subsets studied are shown in Supplemental Figure 2 and Figure 1A, respectively. All groups exhibited comparable frequencies of total and CD56^bright^ NK cells (Figure 1B). However, higher proportion of CD56^dim^CD16^hi^ NK cells was observed in ART individuals (Figure 1B). Interestingly, all PWH exhibited higher frequencies of memory-like NKG2C^+^CD57^+^ NK cells, although significance was only reached for DC, ART, and VIR, compared with HD (Figure 1C). These cells are more likely to represent expanded adaptive NK cells, as previously reported in CMV (39), and lately, in HIV infection (40).

Next, we studied whether NK cells from DC presented a different expression pattern of important receptors for NK activity. For these analyses, we included NK receptors with activating or inhibitory potential upon interaction with ligands found on HIV-infected cells (KIR2DL2/L3 [CD158b], KLRG1, NKG2A, NKG2D, NKp30, and NKG2C), the chemokine receptor CXCR3, and the maturation marker CD57. Expression of selected markers in HD were concordant with previous studies (41). We observed higher expression of the negative receptors CD158b and KLRG1 in ART and VIR groups relative to HD, DC, and AC in total CD56^+^ and CD56^dim^CD16^hi^ NK cells (Figure 1D and Supplemental Figure 3A). By contrast, higher frequencies of NKG2A^+^ and NKp30^+^ NK cells were observed in HD and AC, in both total CD56^+^ and CD56^dim^CD16^hi^ NK cell subsets, compared with ART and VIR groups (Figure 1D and Supplemental Figure 3A). While NK cells from DC generally presented similar receptor expression patterns relative to those from HD and AC groups, DC displayed higher expression of the activating receptor NKG2C in all 3 NK cell subsets (Figure 1D and Supplemental Figure 3, A and B). No remarkable differences were observed in the expression of CXCR3, NKG2D, and CD57 receptors among DC and other groups in both total CD56^+^ and CD56^dim^CD16^hi^ NK cells (Figure 1D and Supplemental Figure 3A). By contrast, increased CXCR3 expression was observed in ART compared with DC and VIR groups in CD56^bright^ NK cells (Supplemental Figure 3B).

Next, we focused on the memory-like NKG2C^+^CD57^+^ NK cell population. For consistency, only samples with expanded NKG2C^+^CD57^+^ NK cells (>5% frequency) were analyzed. Comparative analysis revealed no statistical differences in the expression levels of CD158b, CXCR3, NKG2D, and NKp30 across the distinct study cohorts (Figure 1E). Consistent with the other NK cell subsets, a trend toward elevated frequencies of KLRG1^+^ memory-like NK cells was observed in ART and VIR groups compared with HD, DC, and AC (Figure 1E). Furthermore, within the DC group, heightened frequencies of NKG2A^+^ memory-like NK cells were found compared with ART (Figure 1E).

Overall, our study reveals 2 important aspects. The first is that distinct phenotypic variations are observed within NK cell populations among different PWH groups, underscoring the heterogeneity of the NK cell repertoire during HIV infection. Second, the expression of the different NK cell receptors within the distinct NK cell subsets observed in DC is similar to those observed in AC, and in HD, except for the memory-like NKG2C^+^CD57^+^ subset of cells, which was enriched in NK cells from the DC cohort. Moreover, within the memory-like NK cell subset, the expression of NKG2A was elevated compared with other groups, indicating their potential to regulate their functional responses (42). These identified markers of NK cell populations represent a distinctive hallmark of ECs who spontaneously control HIV for prolonged periods without virological and immunological progression.

### NK cells from individuals with DC exhibit reduced natural cytotoxicity, but enhanced ADCC activity against HIV-infected cells.

**Figure 2:**
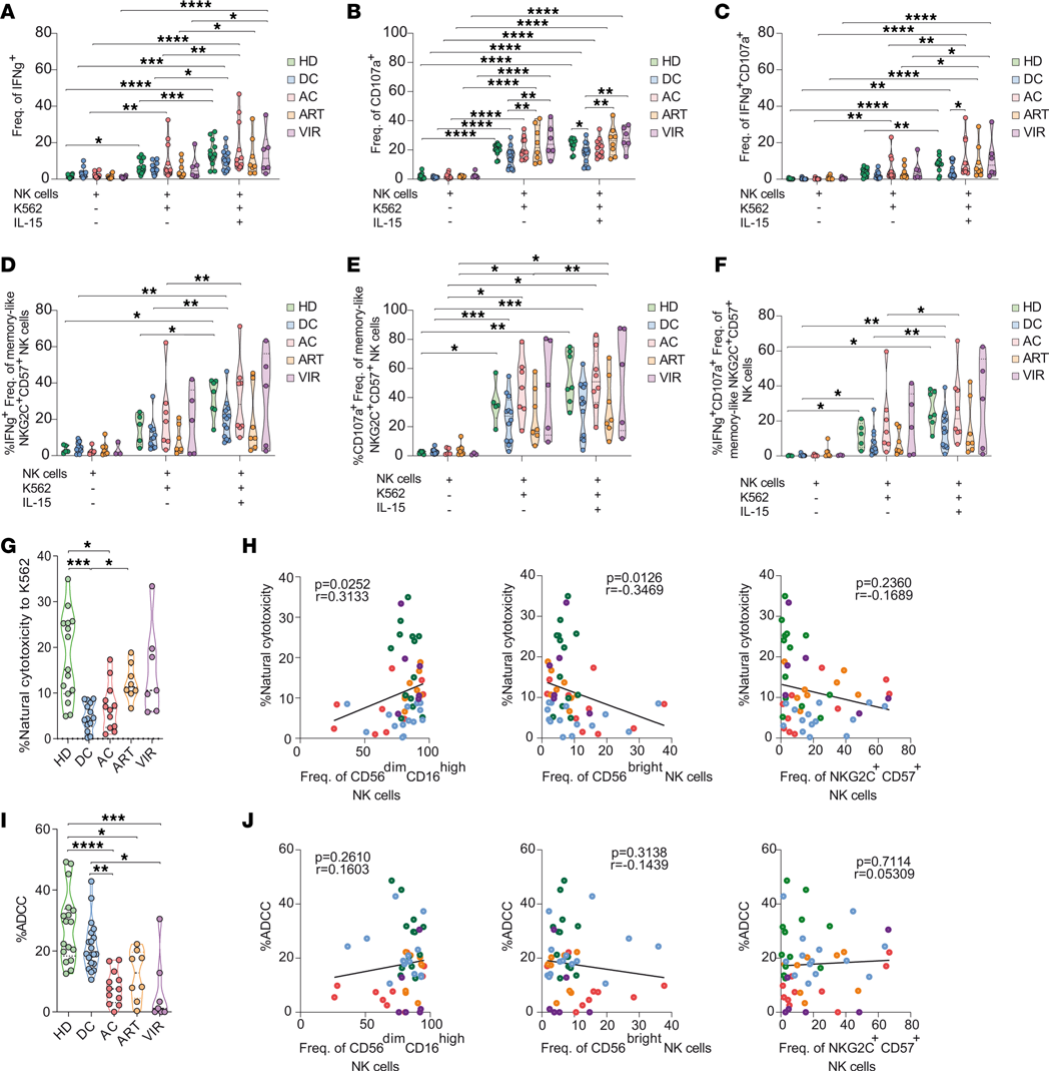
Functional profile of NK cells in ECs. NK cell activation and cytotoxicity subsequent to stimulation were evaluated by study group. The percentages of (**A**) IFN-γ^+^, (**B**) CD107a^+^, and (**C**) polyfunctional IFN-γ^+^CD107a^+^ within the CD56^+^ NK cell population were determined in basal conditions, following coculture with K562 target cells, and with additional IL-15 stimulation. Similarly, these metrics were quantified in expanded memory-like NKG2C^+^CD57^+^ NK cells (frequency >5%): the percentages of (**D**) IFN-γ^+^, (**E**), CD107a^+^, and (**F**) polyfunctional IFN-γ^+^CD107a^+^ NK cells after stimulation were evaluated. (**G**) Violin plots depicting the natural cytotoxicity exhibited by CD56^+^ total NK cells from the different study groups following coculture with K562 cells. (**H**) Spearman’s correlations between natural cytotoxic responses and the frequency of distinct NK cell subsets (left to right: CD56^dim^CD16^hi^ NK cells, CD56^bright^ NK cells, and NKG2C^+^CD57^+^ NK cells). (**I**) Violin plots showing the ADCC activity mediated by CD56^+^ total NK cells against HIV-expressing cells by study group. (**J**) Spearman’s correlations between ADCC responses and the frequency of different NK cell populations (left to right: CD56^dim^CD16^hi^ NK cells, CD56^bright^ NK cells, and NKG2C^+^CD57^+^ NK cells). For violin plots, median with range is represented. *P < 0.05; **P < 0.01; ***P < 0.001; ****P < 0.0001 by repeated measures 2-way ANOVA followed by Tukey’s multiple-comparison test (**A**–**F**) or Kruskal-Wallis test (**G** and **I**).

Next, we aimed to directly elucidate the functional signatures of NK cells in all study groups. We focused on the functional response of DC participants, as they represent a model for a functional cure for HIV. We performed NK cytotoxicity assays against the MHC-devoid K562 cell line, using NK cells that were either unstimulated or primed with IL-15 (gating strategy shown in Supplemental Figure 4A). NK cell degranulation (CD107a) and IFN-γ secretion were measured upon stimulation (Supplemental Figure 4B). Although all study groups showed significant IFN-γ production and degranulation activity against target cells upon stimulation, reduced degranulation responses were observed in total CD56^+^ NK cells from DC compared with HD, ART, and VIR groups (Figure 2, A–C). Similarly, increased IFN-γ production and degranulation were observed in CD56^dim^CD16^hi^ and CD56^bright^ NK cell subsets upon stimulation in all study groups (Supplemental Figure 5, A and B). Notably, the expanded memory-like NK cell population (>5% frequency) showed, in general, higher ability to produce IFN-γ and CD107 upon stimulation, compared with total CD56^+^ NK cells (Figure 2, A–F). A tendency toward decreased degranulation responses was observed within the memory-like NK cell population in DC, exhibiting diminished IFN-γ production, degranulation, and polyfunctional responses (IFN-γ and CD107a expression) compared with HD (Figure 2, D–F).

Subsequently, we evaluated the intrinsic natural cytotoxicity and ADCC responses of NK cells. Natural cytotoxicity was assessed by quantifying their killing capacity against MHC-devoid K562 cell targets. Cell killing was measured by a viability dye–based flow cytometric analysis (Supplemental Figure 6A). ADCC activity of NK cells toward HIV-infected cells was evaluated by coculturing ACH-2 cells with isolated NK cells from the different study groups in the presence of plasma from a viremic HIV^+^ participant containing a pool of antibodies targeting the gp120 protein. Cell killing was determined by the lack of detection of the viral protein p24 in infected cells by flow cytometry (Supplemental Figure 6B).

Notably, NK cells from DC individuals exhibited a significantly reduced cytotoxic response toward K562 target cells (median killing = 4.46%), in contrast with HD (median killing = 15.10%) and ART groups (median killing = 11.30%) (Figure 2G). Specifically, NK cells from DC demonstrated an approximate reduction of 30% in their ability to eliminate target cells compared with HD (Figure 2G). Importantly, and consistent with prior literature (43–45), our findings reveal a positive association between the frequency of CD56^dim^CD16^hi^ NK cells and natural cytotoxic responses, while a negative correlation is observed with CD56^bright^ frequencies (Figure 2H). Of note, no significant associations were found between natural cytotoxic responses and the frequency of memory-like NK cells (Figure 2H), nor between cytotoxic responses and the expression levels of NKG2D (Supplemental Figure 7A), a receptor for which K562 cells express high levels of ligand.

We next evaluated the adaptive ADCC activity of NK cells toward HIV-infected cells. NK cells from DC performed strong ADCC responses (median ADCC = 19.09%), with an overall capacity similar to the one observed for HD (median = 29.63%), for which higher variation was observed (Figure 2I). Defective ADCC was observed for AC and VIR NK cells (median_AC_ = 7.58%, median_VIR_ = 1.18%), which showed significantly reduced activity compared with HD and DC (Figure 2I). Similarly, ADCC from NK cells derived from the ART group was reduced relative to HD (median_ART_ = 12.82%) (Figure 2I). Importantly, the enhanced capacity of NK cells from DC to kill via the ADCC mechanism was not related to the direct abundance of CD56^dim^CD16^hi^ NK cells, the subset primarily implicated in ADCC responses, nor to the frequencies of memory NK cells (Figure 2J). Furthermore, since CD16 expression levels on the surface of NK cells might directly affect ADCC activity, we evaluated differences between groups and found that DC had similar CD16 expression levels compared to other cohorts, and this expression did not correlate with ADCC (Supplemental Figure 7B).

Overall, all study groups exhibited elevated frequencies of activated NK cells, marked by the secretion of soluble effector molecules, such as IFN-γ and CD107a, upon stimulation. However, DC individuals exhibited a tendency toward a limited production of these molecules compared with other groups, particularly within the memory-like NK cell subset; however, these differences did not reach statistical significance. These functional changes in NK cells from DC translated into lower natural cytotoxicity. In contrast, DC NK cells demonstrated a potent ADCC response against HIV-infected cells, which was not related to the presence of any major NK subset, suggesting a distinctive feature of NK cells from DC individuals.

### A distinctive memory-like NK population in DC is associated with enhanced ADCC responses against HIV-infected cells.

**Figure 3:**
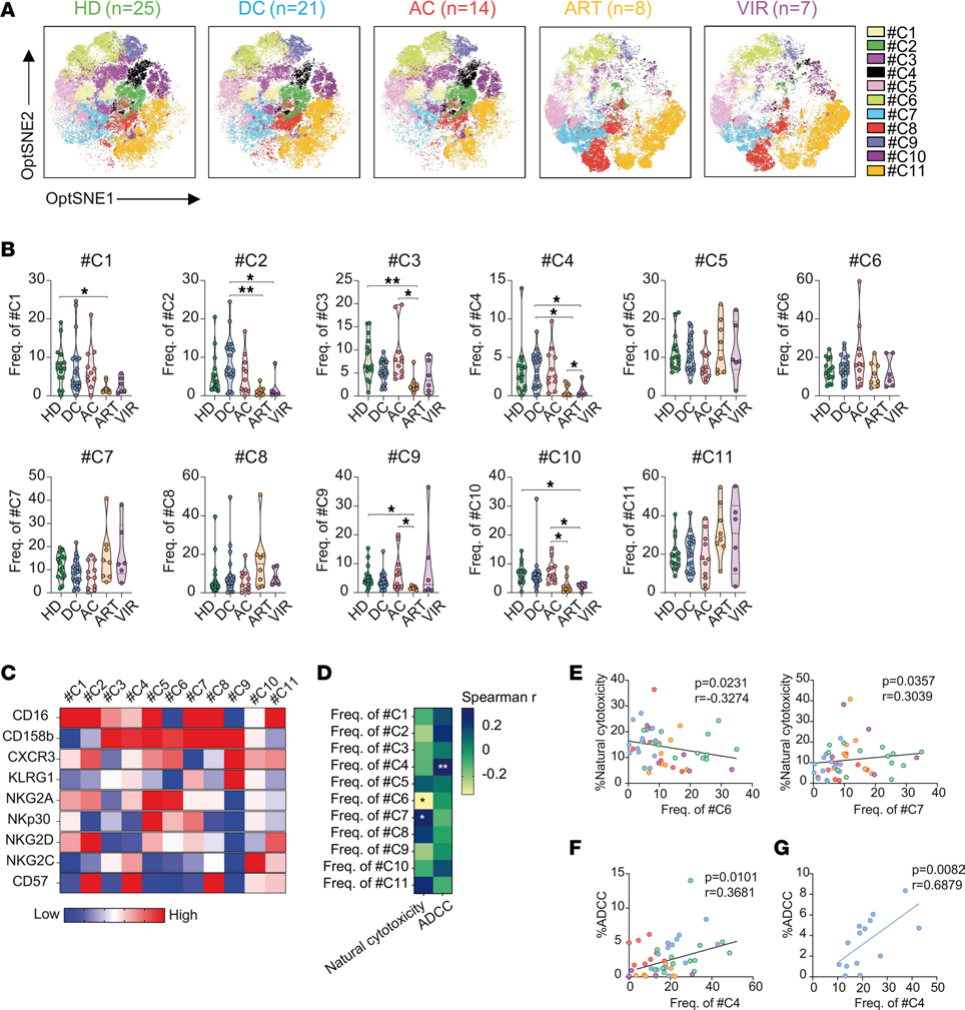
Association between NK cell phenotype and functional responses in EC. (**A**) Optimized t-distributed stochastic neighbor embedding (opt-SNE) representation of distinct NK cell clusters, identified through dimensionality reduction based on the expression of the array of distinct NK cell receptors (CD16, CD158b, CXCR3, KLRG1, NKG2A, NKp30, NKG2D, NKG2C, and CD57), by study group (left to right: HD, DC, AC, ART, and VIR). (**B**) Violin plots showing the frequency of each NK cell cluster in CD56^+^ total NK cells by study group. (**C**) Heatmap depicting the normalized median expression of the selected phenotypic NK cell markers within the cell clusters identified in Figure 3A. (**D**) Correlation matrix depicting Spearman’s correlations between the frequency of NK cell clusters identified by dimensionality reduction based on the expression of phenotypic markers and functional responses. (**E**) Spearman’s correlations between the frequency of clusters C6 and C7 and natural cytotoxic responses in all study groups. (**F** and **G**) Spearman’s correlations between the frequency of C4 and ADCC responses in (**F**) all study groups and (**G**) within the DC group. Graphs represent medians and ranges. Each dot represents 1 individual of a specific cohorts, indicated by color code (HD in green, DC in blue, AC in red, ART in orange, VIR in purple). Statistical comparisons were performed using the Kruskal-Wallis test. *P < 0.05; **P < 0.01.

Next, to uncover novel NK cell subsets linked with the functional responses observed in DC individuals, we explored the landscape of the different NK cell populations by reduction of dimensionality analysis. Unsupervised clustering analysis via the FlowSOM algorithm allowed us to identify 11 different NK cell clusters with an individual pattern of expression of the selected receptors (Figure 3A). Importantly, we identified C2 and C4 as clusters of interest, as they were enriched in individuals with DC (Figure 3B). These clusters comprised cells expressing CD16, an important receptor to mediate ADCC, together with CXCR3, NKG2A, and CD57 (Figure 3C). Cluster C2 exhibited high expression of NKG2D, an activating receptor associated with ADCC responses, whereas cluster C4 demonstrated low expression of this marker (Figure 3C). By contrast, C4 additionally exhibited CD158b and NKG2C expression, representing a unique memory-like NK cell phenotype (Figure 3C and Supplemental Figure 8A).

Subsequently, we examined the relationship between functional NK cell responses and the frequency of the distinct NK cell clusters (Figure 3D). A significant negative correlation was observed between cluster C6 and natural cytotoxic responses (*r* = –0.33, *P* = 0.02), while a positive correlation was noted between cluster C7 and natural cytotoxicity (*r* = 0.30, *P* = 0.03) (Figure 3, D and E). Notably, there were no significant differences in the frequency of these clusters among study groups (Figure 3B). Both clusters prominently expressed CD158b and NKp30, while NKG2C and CD57 markers were either low or absent (Figure 3C). However, cluster C7, which associated with higher natural cytotoxicity, displayed elevated levels of CD16 and KLRG1, distinguishing it from cluster C6, which was defined by the coexpression of CXCR3 and NKG2A (Figure 3C). In addition, cluster C2 exhibited a trend for a positive correlation with ADCC responses, although no statistical significance was achieved (Figure 3D). Importantly, cluster C4, predominantly composed of memory-like NK cells expressing NKG2A and CXCR3, exhibited a robust association with ADCC responses (Figure 3, D and F). Remarkably, this correlation was particularly strong within the DC group (*P* = 0.0082, *r* = 0.68) (Figure 3G). Importantly, although both DC and AC showed similar proportions of C4, we demonstrated that only DC had an enhanced ability to mediate ADCC (Figure 2I). We hypothesize that this could be explained, at least in part, by the significantly higher expression of NKG2C observed in C4 from DC, which is directly related to the ability to perform ADCC (Supplemental Figure 8, A and B).

Overall, we identified different NK populations associated with specific functions. A CD16^+^CD158b^+^NKp30^+^NKG2D^dim^KLRG1^dim^ population correlated with better natural cytotoxicity activity, while a unique memory-like NK cell population was associated with enhanced ADCC responses, especially in DC. This memory-like NK subset is characterized by the expression of CXCR3, indicating its potential to migrate to inflamed tissues, and NKG2A, an inhibitory receptor that identifies early-differentiated NK cells. This identified NK cell population represents a characteristic feature of individuals exhibiting natural HIV control.

### Memory-like NK cells coexpressing NKG2A and CXCR3 exhibit heightened ADCC responses against HIV-infected cells.

**Figure 4:**
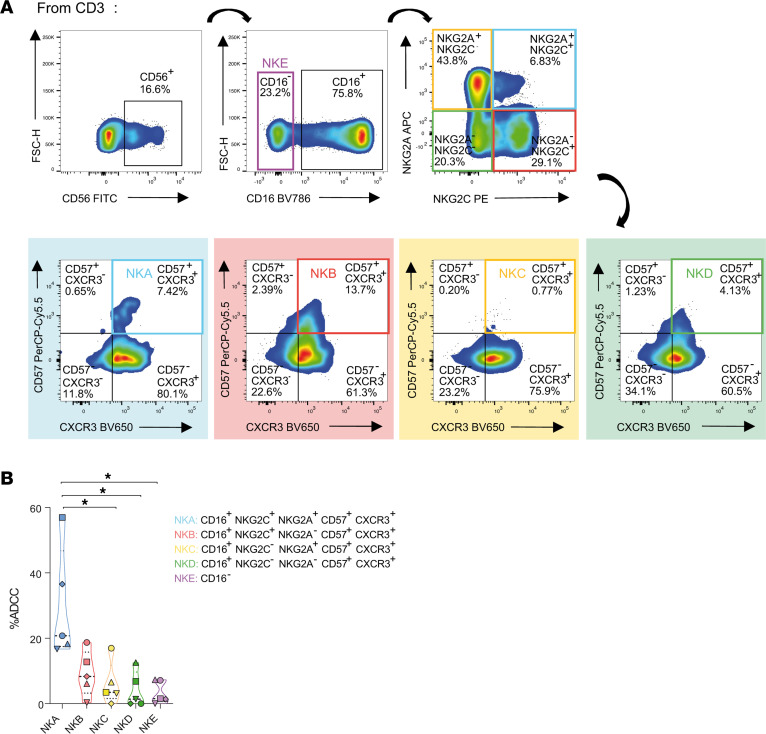
NK cells from DC enriched in specific receptors exhibit an increased ability to kill HIV-infected cells. Distinct NK cell subsets were isolated from *n* = 5 DC individuals (median CD4^+^ T cell count = 1030 cells/μL; median viral load <40 copies HIV-1 RNA/mL) using FACS based on selected receptors, and assessed for ADCC responses. (**A**) Gating strategy of FACS-isolated NK cell populations based on the expression of CD16, NKG2A, NKG2C, CD57, and CXCR3. Five populations were identified: NKA (CD16^+^NKG2A^+^NKG2C^+^CD57^+^CXCR3^+^), NKB (CD16^+^NKG2A^–^NKG2C^+^CD57^+^CXCR3^+^), NKC (CD16^+^NKG2A^+^NKG2C^–^CD57^+^CXCR3^+^), NKD (CD16^+^NKG2A^–^NKG2C^–^CD57^+^CXCR3^+^), and NKE (CD16^–^). (**B**) Violin plot depicting the ADCC activity mediated by each specific NK cell population based on the marker expression presented in **A**. Graphs include group medians and ranges. Statistical comparisons were performed using Friedman’s test followed by Dunn’s multiple-comparison test. *P < 0.05.

Next, we aimed to validate whether the memory-like NK cell population previously identified and linked to ADCC responses indeed displayed enhanced ADCC activity. Given the unusual coexpression of NKG2C and NKG2A on NK cells, we initially evaluated the expression of these markers in NK cells across all study groups by flow cytometry. As expected, the fraction of circulating NK cells expressing both markers was low (<15% of total CD56^+^ NK cells) (Supplemental Figure 9). Due to cell number limitations, ADCC assays were only performed on DC samples. We performed a functional ADCC assay using samples from 5 DC individuals. Distinct NK cell populations were isolated by FACS, based on the expression of selected receptors (Figure 4A). The cell numbers obtained after cell sorting are shown in Supplemental Table 2. Briefly, CD16^+^ cells derived from CD56^+^ NK cells were classified based on the expression of NKG2A, NKG2C, CD57, and CXCR3. NK cell classification included NKA (NKG2A^+^NKG2C^+^CD57^+^CXCR3^+^), NKB (NKG2A^–^NKG2C^+^CD57^+^CXCR3^+^), NKC (NKG2A^+^NKG2C^–^CD57^+^CXCR3^+^), and NKD (NKG2A^–^NKG2C^–^CD57^+^CXCR3^+^). CD16^–^ NK cells were designated as NKE. NK cells were then incubated with HIV^+^ plasma and cocultured with ACH-2 HIV-infected cells for 4 hours. We calculated cell killing as the reduction in the percentage of virally infected cells.

NKA, characterized by the coexpression of NKG2A and NKG2C, exhibited a more robust ADCC response against HIV-infected cells than any other NK population (median ADCC = 20.78%), especially compared with NK cells lacking CD16 expression (NKE, median ADCC = 1.54%), NKC (median ADCC = 3.49%), and NKD (median ADCC = 1.38%) (*P* < 0.05) (Figure 4B). In general, NKA exhibited the ability to eliminate approximately 50% more HIV-infected cells than the other NK cell subpopulations (median ADCC for NKB = 8.33%, NKC = 3.49%, NKD = 1.38%). Altogether, our findings suggest that the expression of the receptors CD16, NKG2A, NKG2C, CD57, and CXCR3 on NK cells identifies a memory-like population with enhanced capacity for killing HIV-infected cells through ADCC.

### Single-cell transcriptional analyses identifies NKA as a population with a unique transcriptional signature linked to increased NK cell effector functions, migration, and antiviral responses.

Finally, to elucidate the molecular expression patterns associated with enhanced ADCC responses in DC individuals, we conducted single-cell RNA-seq analyses. We aimed to characterize and identify differentially expressed genes within NKA, particularly in comparison to conventional adaptive NK cells. For comparative purposes, we analyzed 3 related NK cell populations previously delineated in the functional ADCC analysis (NKA, NKB, and NKC). All 3 populations were characterized by high protein expression of CD16, CXCR3, and CD57. A total of 7226, 7354, and 8180 cells were sequenced for NKA, NKB, and NKC, respectively. NKA exhibited 2618 genes and 28,800 reads per cell, NKB showed 2516 genes and 26,791 reads per cells, and NKC displayed 2584 genes and 24,379 reads per cell. To check whether the data were appropriate for downstream analysis, different types of quality controls were performed, including the quality control of raw data (FastQC), the alignment and quantification of reads to described mRNAs (Cell Ranger), the number of transcripts per cell, and the percentage of counts coming from different transcript types, as previously described (46–48).

**Figure 5:**
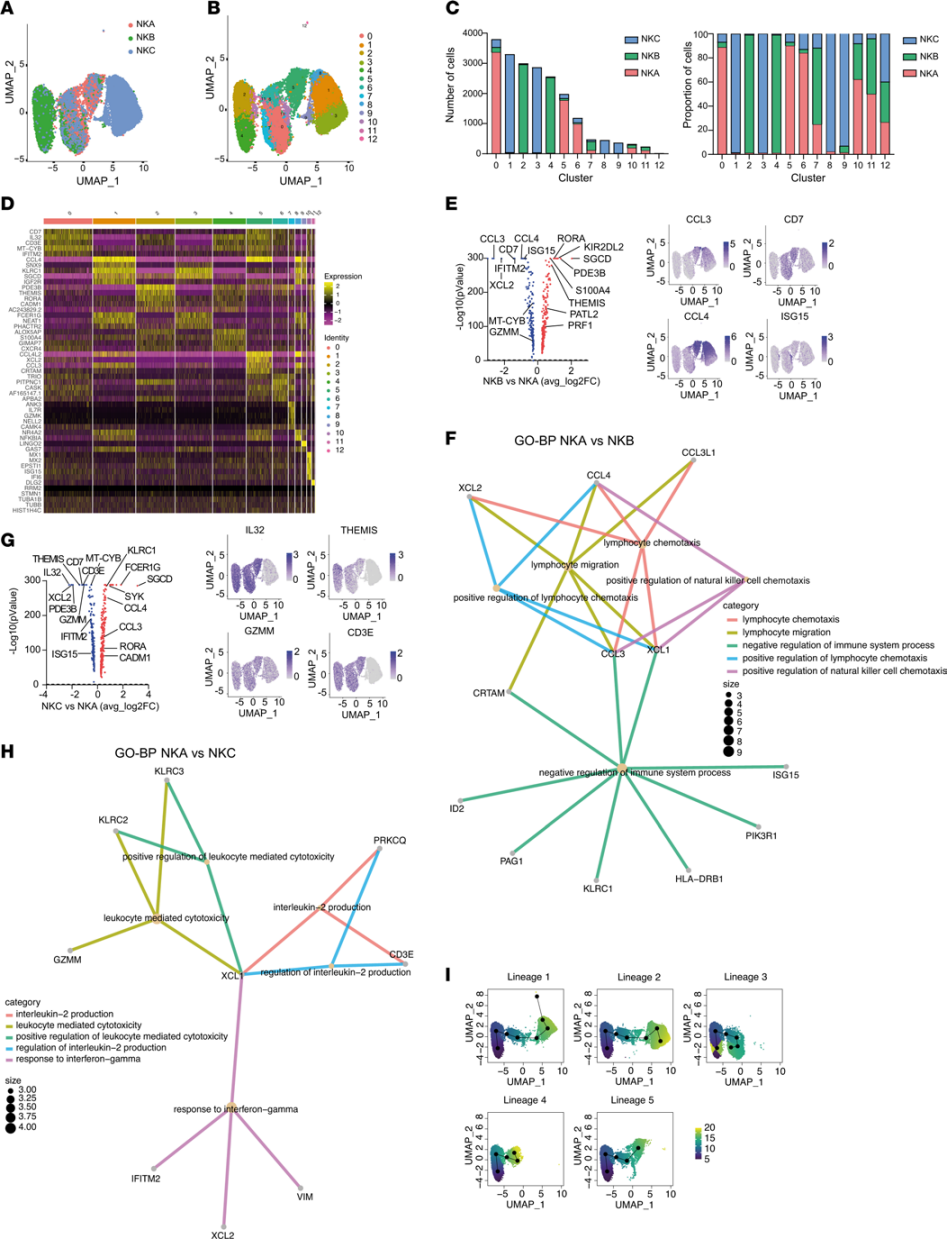
Unique transcriptional signatures define ADCC-mediating NK cells from DC. Gene expression analysis by single-cell RNA-seq. (**A**) UMAP visualization of NK cell populations sorted from DC individuals (NKA in red, NKB in green, NKC in blue). (**B**) UMAP visualization of 13 distinct NK cell clusters identified from NK cell populations sorted by unsupervised hierarchical clustering. (**C**) Number of cells per cluster and sample as counts (left) or proportions (right). (**D**) Heatmap depicting the top 5 genes most differentially expressed (upregulated) in each NK cell cluster. (**E**) The top panel shows a volcano plot illustrating the differentially expressed genes between NKB and NKA subsets. Genes significantly overexpressed in NKB compared with NKA are highlighted in red, while those underexpressed in NKB relative to NKA are shown in blue. The bottom panel presents representative UMAP plots depicting the expression of genes upregulated in NKA compared with NKB. (**F**) Significant canonical pathways predicted by Gene Ontology Biological Process analysis (GO-BP) of differentially expressed genes in NKA versus NKB. (**G**) The top panel presents a volcano plot illustrating the differentially expressed genes between NKC and NKA subsets. Genes significantly overexpressed in NKC relative to NKA are shown in red, while those underexpressed in NKC compared with NKA are depicted in blue. The bottom panel displays representative UMAP plots highlighting the expression of genes upregulated in NKA compared with NKC. (**H**) Significant canonical pathways predicted by GO-BP of differentially expressed genes in NKA relative to NKC. (**I**) Differentiation trajectory analysis of NK cell clusters, illustrating the progression and differentiation pathways of each population. Five distinct lineages were identified. The pseudotime inference for each NK cell cluster is presented, with UMAP plots color coded by inferred lineages. The scale indicates the maturation state, ranging from yellow (least mature) to dark blue (most mature).

To delineate the heterogeneity of ADCC-mediating NK cells, we compared the transcriptional landscape of the distinct NK cell subsets by projecting cells into 2 dimensions by uniform manifold approximation and projection (UMAP) analysis. UMAP analysis unveiled NKB and NKC cell subsets as 2 separate populations, while NKA cells represented an intermediate state between both (Figure 5A). Unsupervised clustering analyses via shared nearest neighbor (SNN) modularity optimization–based clustering algorithm resulted in the identification of 13 distinct NK cell clusters (Figure 5B). Of note, clustering was not significantly influenced by the cell cycle (Supplemental Figure 10A). Differential expression analysis identified conserved markers for each cluster, irrespective of the NK cell population (Supplemental Table 3). For optimization purposes, differential expression analysis only included genes with a minimum log_2_(fold change) above 0.25 and expressed in at least 10% of cells in either of the 3 populations tested.

The NKA population, defined by the coexpression of NKG2C and NKG2A, encompassed clusters C0, C5, C6, and C10 (Figure 5, B and C). Cluster C0, the most prevalent within the NKA population, exhibited a transcriptional profile marked by the upregulation of genes indicative of adaptive NK cell signatures, including *IL32* and *CD3E*, while notably lacking *FCER1G* (30) (Figure 5D). Additionally, it exhibited *MTCYB* expression, signifying elevated metabolic demands, and *IFITM2* expression, indicating an enhanced antiviral response (Figure 5D). Moreover, cluster C5 displayed a transcriptomic enrichment of genes encoding soluble factors integral to NK cell effector functions, such as *CCL3*, *CCL4*, and *CCL4L2*, as well as *XCL2*, a chemokine characteristic of naive CD56^bright^ NK cells involved in the activation of the immune system and chemotaxis (49) (Figure 5D). Additionally, cluster C6 demonstrated upregulation of genes implicated in actin cytoskeleton remodeling and lipid metabolism, including *TRIO*, *PITPNC1*, *CASK*, and *APBA2* (Figure 5D). Importantly, cluster C10 exhibited an upregulation of genes coding for IFN-induced proteins associated with the NK cell antiviral response, specifically *MX1*, *MX2*, *ISG15*, and *IFI6* (Figure 5D). The NKB subset, characterized by high expression of NKG2C, comprised clusters C2, C4, and C7 (Figure 5, B and C). Clusters C2 and C4 presented the canonical transcriptional profile of adaptive NK cells, exhibiting upregulated expression of *IL32* and *CD3E*, while lacking *FCER1G* and *CD7* expression (Figure 5D). Furthermore, these clusters showed enhanced expression of genes associated with differentiation, maturation, and effector functions (*THEMIS*, *RORA*, *PDE3B*, and *CADM1*) (Figure 5D). Notably, cluster C7 was defined by the upregulation of genes involved in cell adhesion, activation, and differentiation (*ANK3*, *IL7R*, *GZMK*, and *NELL2*) (Figure 5D). By contrast, the NKC subset, defined by elevated NKG2A expression, comprised clusters C1, C3, C8, and C9 (Figure 5, B and C). Clusters C1 and C3 were characterized by an upregulation of genes involved in migration and cytokine production (*SNX9*, *SGCD*, *IGF2R*, and *FCE1RG*) along with chemokines (*CCL3* and *CCL4*) (Figure 5D). Additionally, clusters C8 and C9 exhibited upregulated expression of genes regulating NK cell activation (*NFKBIA* and *LINGO2*) (Figure 5D).

Despite exhibiting adaptive NK cell traits, a direct comparison of the transcriptomic profile of the NKA subset with the conventional memory-like NKB population unveiled a heightened expression of CD7, a marker typically downregulated in adaptive NK cells (Figure 5E). NKA cells also presented elevated gene expression levels of *CCL3*, *CCL4*, and *XCL2* chemokines, known for their pivotal role in NK cell activation and recruitment (Figure 5E and Supplemental Figure 10, B and C). Additionally, NKA exhibited heightened expression of *ISG15*, an IFN-stimulated gene (ISG) pivotal in inducing IFN-γ in the antiviral response, and *MTCYB*, a regulator of mitochondrial biogenesis and ATP synthesis, implicated in the secretion of cytolytic granules and cytokines (50) (Figure 5E). Of note, compared with NKB, NKA showed downregulation of *RORA*, a transcription factor involved in ILC2 differentiation (51) (Figure 5E and Supplemental Figure 10, B and C). Furthermore, Gene Ontology (GO) analysis revealed that the NKA subset exhibited increased pathways related with enhanced lymphocyte chemotaxis, migration, and cell regulation (Figure 5F).

Moreover, relative to the NKC subset, the NKA population displayed increased expression of *IL32* and *THEMIS*, which are involved in the regulation of the NK cell cytolytic response and memory NK cell differentiation (Figure 5G and Supplemental Figure 10, D and E). Additionally, compared with NKC, NKA exhibited the upregulation of *GZMM* and *CD3E* genes that are implicated in NK cell cytotoxicity (Figure 5G and Supplemental Figure 10, D and E) and compatible with the upregulation of pathways related to cell cytotoxicity, IL-2 production, and response to IFN antiviral response (Figure 5H). In contrast with NKC, NKA exhibited downregulation of *CCL3* and *CCL4* chemokines, alongside *FCER1G*, which is associated with an NK-mediated regulation of the CD8^+^ T cell response in virus control (52) (Figure 5H). Importantly, compared with both NKB and NKC populations, NKA exhibited elevated expression of *XCL2*, *CD7*, *MTCYB*, *IFITM2*, and *ISG15* that are associated with NK cell migration, differentiation, cell metabolism, and antiviral response (Figure 5, E–H).

Finally, we performed a gene expression trajectory analysis to elucidate the continuum of dynamic changes occurring within these distinct NK cell clusters. A total of 5 distinct lineages, defined as ordered sets of cell clusters sharing a common starting or ending point, were identified using Slingshot (Bioconductor) (Supplemental Table 4 and Figure 5I). Additionally, for each identified lineage, pseudotimes were determined, representing the variable indicating the transcriptional progression of each cell toward the terminal state (Figure 5I). Notably, irrespective of their origin, cells from all lineages transitioned through the NKA population, specifically cluster C10, and consistently ended within cluster C4, highly predominant in the NKB population. This observation suggests that all NK cell subsets studied transition into an intermediate population, distinguished by the expression of NKG2A and NKG2C. Furthermore, the final differentiation stage of all these identified lineages constitutes cells with a differentiated and adaptive NK cell signature (Supplemental Table 4 and Figure 5I).

Altogether, our data support the notion that NKA cells expressing NKG2C and NKG2A, which are associated with higher ADCC responses in DC, represent a transitional state between a less differentiated population (NKC) and a more mature and adaptive state (NKB). Importantly, these cells are characterized by upregulation of specific genes associated with enhanced effector functions, cell migration, metabolism, and antiviral responses. Overall, our data support the potential of NKA cells to mediate strong cytotoxic pressure against HIV.

## Discussion

Despite the success of ART, completely eliminating HIV from PWH remains extremely challenging due to the establishment of long-lived latent reservoirs (53). The ability of a small number of PWH to resist disease progression and remain disease free for prolonged periods makes these individuals attractive candidates to study and improve our understanding of the HIV pathogenesis. Although full viral elimination is not achieved, ECs present undetectable levels of HIV replication by clinical assays, and therefore, represent a model of spontaneous functional cure (4, 6). Major determinants of HIV control affect HLA class I peptide presentation, implying a central role in CTL responses (15, 20, 54, 55). However, the role of their innate immune system in controlling HIV remains unclear. In the present study, we have characterized the phenotype, functional attributes, and transcriptional signature of the NK cell repertoire of ECs. We report that certain NK cell populations, especially cells with maturation and memory-like attributes, are more characteristic of people with DC. Moreover, NK cells from these individuals are more refractory to cell stimulation and show a reduced natural cytotoxic activity, while showing an extraordinary ability to kill by the adaptive ADCC mechanism. Importantly, the distinctive ability to eliminate HIV-infected cells might be attributable to the presence of NK cells with a unique transcriptional landscape, associated with increased antiviral response. Thus, we add evidence for the importance of NK cells in HIV control.

Numerous changes within the NK cell repertoire have been previously associated with HIV infection, including an increase in inhibitory receptors, such as KIR CD158b, and a switch from inhibitory (NKG2A) to activating NKG2 receptor expression (NKG2C) (27, 56, 57). Concordantly, our study confirms these changes in NK cell populations from ART and VIR groups compared with HD. Interestingly, the distribution of these markers on DC was similar to HD, except for NKG2C, which was significantly increased in DC. This is noteworthy since this receptor is, in part, providing activating signaling upon engaging its ligand, HLA-E, expressed on the target cell. In addition, in our study NKG2C^+^CD57^+^ NK cells appeared to be present in PWH, particularly expanded in DC and ART individuals. This phenotype has been associated with memory-like features, particularly in the context of CMV infection (36). Moreover, CD57 is expressed in mature NK cell subsets that appear to be highly cytotoxic and its expression is consistently associated with better outcomes in cancer and autoimmune diseases (58). Of note, in the context of HIV infection, a recent study identified CD57^+^NKG2C^+^ NK cells as the predominant ADCC effector subset capable of targeting HIV-infected CD4^+^ T cells in the presence of broadly neutralizing antibodies (38, 59). This is in agreement with the association between NKG2C^+^ memory-like NK cells and the control of viral replication in a recently reported posttreatment controller (60).

Importantly, our study shows that although these memory-like NK cells were observed in PWH, only specific memory-like NK populations, primarily found in DC, were related to better ADCC outcomes. This indicates that the mere presence of these CD57^+^NKG2C^+^ NK cells does not necessarily translate into improved NK functionality. In this regard, we found that important receptors involved in NK cell differentiation and regulation of their function, such as CD16, CD57, CXCR3, and NKG2A, were enriched in DC. This observation is of significance, as it suggests that NK cell subsets exhibiting varying differentiation states may potentially impact the course of HIV infection within the distinct study cohorts. Remarkably, expanded subsets in DC exhibited elevated NKG2A levels, coexpressing NKG2C, an unusual phenomenon considering their opposing functions, while binding the same ligand. NKG2A, associated with inhibitory signaling, typically appears on less mature NK cell subsets, whereas NKG2C, linked to activating signals, characterizes more mature populations (61, 62). Coexpression of both markers is infrequent in healthy donor peripheral blood NK cells, but has been linked to reduced cytotoxic and proinflammatory responses, suggesting a role in NK cell function regulation (62, 63). Furthermore, it has been reported that despite the presence of HLA-E on HIV-infected cells, the NK cell subset most likely to respond to them is characterized by the expression of NKG2A (64). Thus, we hypothesize that coexpression of NKG2A and NKG2C in DC-derived NK cells may denote a partially differentiated phenotype capable of balancing activating responses, while maintaining a high potential to kill HIV-infected cells. In addition, these populations also showed an upregulation of the chemokine receptor CXCR3, which may facilitate the efficient recruitment of these cells to tissues (65).

Currently, it is challenging to ascertain whether the specialized expanded memory NK cells primarily observed in individuals with DC have arisen due to a prior CMV reactivation or, as recently suggested, following acute HIV infection (40). It is worth noting that due to sample limitations, we were unable to determine the CMV serological status of our participants. However, numerous studies involving PWH have consistently indicated a significantly higher seroprevalence of CMV in this group compared with the uninfected population, with antibody levels consistently detected in more than 90% of PWH (66–68). Hence, further studies are required to elucidate the precise timing of the generation of these NK cells and the mechanisms underlying their expansion.

HIV control is associated with ADCC, where specific antibodies bind to viral antigens on the surface of infected cells, and by Fc receptor engagement, which recruits NK cells to eliminate HIV-infected targets. The importance of Fc-mediated functions was highlighted by the partially successful RV144 HIV vaccine trial, in which ADCC antibodies were associated with protection from HIV (69). Previous studies described impairment of the NK-mediated ADCC activity during early HIV infection, indicative of premature virus-mediated NK cell dysfunction (57, 70). In contrast, individuals with natural HIV control exhibited elevated levels of ADCC antibodies in plasma, particularly against gp120 and envelope antigens (31, 71–73). In fact, ECs that presented an HIV reservoir size below the limit of detection had significantly higher ADCC activity than those with a detectable HIV reservoir size (31). While supporting the role of ADCC in HIV control in PWH displaying natural viral suppression, these studies predominantly investigated the functionality and diversity of ADCC antibodies in an in vitro model, employing NK cells from HIV-seronegative donors and CEM target cells. However, to our knowledge, existing studies have provided limited data comprehensively characterizing the ADCC potential of NK cells from HIV ECs (74, 75). Our study demonstrates robust ADCC responses in NK cells from PWH exhibiting spontaneous viral control, comparable to HD, and superior to other PWH groups. It is tempting to hypothesize that ADCC responses in DC are preserved and, therefore, similar to those in HD. However, we believe that the mechanisms behind the increased ADCC in DC differ from those in HD. For instance, differences in the expression of certain markers, notably NKG2C, suggest a distinct mechanism. Thus, these findings support the idea that NK cells in ECs can effectively mediate the clearance of HIV-infected cells via ADCC, underscoring the pivotal role of this mechanism in HIV control.

NK cell activation and subsequent cytotoxicity, independent of ADCC responses, can exert a substantial impact on the regulation of viral replication and HIV control. In our study, we analyzed the complexity of NK cell activation and cytotoxic responses upon stimulation with relevant targets such as K562 cells, which do not express MHC-I molecules. In agreement with previous reports, we observed that NK cells from all PWH groups preserve their cytotoxic potential, including degranulation and IFN-γ secretion upon stimulation (76). However, PWH with long-term spontaneous control of HIV showed reduced capacity to produce functional molecules upon cell stimulation and a decreased ability to kill target cells. This observation is consistent with previous studies showing that NK cell populations in HIV ECs exhibit lower levels of cell activation (77, 78). In fact, NK cells can be activated through TLR stimulation, as evidenced by prior studies using HIV-derived ssRNA (79). Consequently, the reduced expression of activation molecules in HIV ECs may be linked to a decrease in the antigen burden. Still, in the case of the reduced activation in NK cells, we believe that may be the result of the intricate interplay between inhibitory and activating receptors. This balance could potentially redirect NK cell responses, favoring an HIV-specific ADCC response over nonspecific activation. Our results underscore a dynamic response of NK cells during HIV infection, potentially influencing the differential outcomes observed in PWH with distinct levels of viral control.

Single-cell RNA-seq of ADCC-mediating NK cells revealed a unique transcriptional NK cell profile associated with enhanced NK cell metabolism, effector functions, cytokine secretion, migration, and an enhanced antiviral response. Notably, these cells exhibited pronounced upregulation of soluble factors involved in immune modulation (*XCL1* and *XCL2*), cytokines (*IL32*), chemokines (*CCL3* and *CCL4*), and ISGs. *XCL1* and *XCL2*, chemokines synthesized following NK cell activation, play a pivotal role in modulating NK cell activity and promoting antigen presentation by dendritic cells (80). Recent studies have described that these chemokines can inhibit HIV infection by blocking viral attachment and entry by binding to the HIV gag polyprotein (81). In addition, IL-32, a characteristic cytokine of adaptive NK cells, was initially identified as a multifunctional cytokine with inhibitory effects on HIV infection (82). Specifically, IL-32β has been described to dampen inflammation by inducing the expression of antiinflammatory cytokines in various cell types (83). Furthermore, activated NK cells secrete CCL3 and CCL4, which play a crucial role in recruiting various inflammatory cells to sites of inflammation. These cytokines have also been reported to inhibit viral replication by interacting with CCR5, a coreceptor expressed on CD4^+^ T lymphocytes and monocyte-derived macrophages that serves as an entry point for HIV into cells (84, 85). Moreover, the upregulation of ISGs in response to viral infection plays a pivotal role in inhibiting HIV replication by targeting various stages of the viral cycle. Specifically, *ISG15* has been shown to interfere with the ubiquitylation of HIV gag protein, which is essential for HIV budding and release (86). *HERC5* has also been reported to impede HIV assembly through ISGylation of gag (87). Finally, we demonstrated that these cells, characterized by enhanced effector NK cell responses, represent a transitional and intermediate state between CMV-expanded adaptive NK cells and naive NKG2A^+^ NK cells. These cells exhibit upregulated gene expression of canonical CMV-expanded adaptive NK cell markers (*IL32*, *CD3E*, *KLRC*, and lack of *FCER1G*) alongside *CD7* and *XCL2*, markers previously reported to be downregulated in adaptive NK cells (30).

Overall, we found that NK cells from PWH with DC of HIV are enriched in cells with a unique transcriptional profile that can be identified by exclusive phenotypic and functional features associated with HIV infection control. A better understanding of the mechanism of natural resistance to HIV infection may have important implications for the design of new anti-HIV therapeutic strategies based on the particular functional activity of NK cells. Thus, we believe that immunotherapy approaches focused on boosting desirable NK responses, such as the ones reported here, could have potential implications in HIV cure strategies.

### Limitations of the study.

This study has some limitations. First, we did not measure the HIV reservoir, or intact HIV genomes among study samples; however, limited quantities of PBMCs led to prioritization of phenotypic, functional, and transcriptomic assays. Second, the ADCC linked to NKG2C expression in DC may be influenced by prior CMV imprinting in NK cells. Future studies should compare ADCC in participants matched for CMV serostatus. Third, we measured phenotypic and functional markers from one time point in each study participant; longitudinal investigations will be needed to extend this analysis in the future. Fourth, the limited sample size restricts the scope of functional assays performed, reflecting the scarcity of participants within these study groups. Fifth, the ADCC assay employed in this study does not account for the quality of autologous antibodies from participants and their potential impact on ADCC responses. However, we believe that ADCC-mediating antibodies from ECs have been extensively characterized in previous studies. Last, the EC group is heterogeneous, characterized by different definitions based on factors such as the length of follow-up, viremia detection limits, presence/absence of blips, and CD4^+^ T cell counts. This inherent heterogeneity introduces variability in the study results and interpretations. Despite these limitations, we believe that this study represents a relevant contribution to the field of HIV control.

## Methods

### Sex as a biological variable.

Cisgender women and men were included in the study.

### Ethics statement and study samples.

Blood samples for the HD group (*n* = 25) were obtained from the Blood and Tissue Bank, Barcelona, Spain (register number C.0003590). Collected samples were anonymous and untraceable. Blood samples from 2 cohorts of PWH (*n* = 66) were obtained from the HIV unit of the Hospital Universitari Vall d’Hebron: ART (*n* = 24, 2–4 years undetectable viral load) and recently diagnosed VIR (*n* = 18, HIV-1 RNA = 30,000–900,000 copies/mL, CD4^+^ T cell counts = 300–1400). PBMC samples from *n* = 34 HIV ECs were obtained from the Spanish AIDS Research Network (RIS) cohorts of HIV Controllers Study Group (ECRIS) database. Viral load and complete blood count records were obtained from healthcare providers with participant consent (Supplemental Figure 1).

EC samples were selected and classified in 2 different EC phenotypes according to their ability to maintain long-term viral and immunological control. AC (*n* = 13) was defined as 5–10 years of documented HIV control of viral loads below 40 HIV-1 RNA copies/mL and stable CD4^+^ T cell counts in the absence of ART prior to a progressive, but significant, CD4^+^ T cell decline during the patient’s follow-up period, evaluated by linear regression (*P* < 0.05) without subsequent return to stable control. DC (*n* = 21) was defined by maintenance of viral loads below 40 HIV-1 RNA copies/mL and stable CD4^+^ T cell counts over time. Participant characteristics are summarized in Supplemental Table 1.

Due to sample accessibility, phenotypic analysis was conducted on all available samples, while functional analyses were restricted to a subset: *n* = 14 HD, *n* = 14 DC, *n* = 10 AC, *n* = 8 ART, and *n* = 7 VIR.

### Cells lines and reagents.

PBMCs were obtained from peripheral blood samples using density-gradient centrifugation with Ficoll-Hypaque (Lymphoprep, StemCell Technologies). Cells were then cryopreserved in liquid nitrogen in FBS (Gibco/Life Technologies) containing 20% dimethyl sulfoxide (DMSO; Thermo Fisher Scientific) and stored until required for functional studies. The human lymphoblastic leukemia ACH-2 cell line (RRID: CVCL_0138) was obtained through the NIH AIDS Reagent Program, Division of AIDS, National Institute of Allergy and Infectious Diseases, from Thomas Folks. The human myeloid leukemia K562 cell line (RRID: CVCL_0004) was purchased from Sigma-Aldrich. Cells were cultured in RPMI 1640 medium supplemented with 10% FBS, 100 U/mL penicillin, and 100 μg/mL streptomycin (Life Technologies) (R10; control medium), and maintained at 37°C in a 5% CO_2_ incubator. NK cells were isolated from PBMCs using a commercial kit (MagniSort Human NK cell Enrichment Kit, eBioscience). Recombinant human IL-15 was purchased from Miltenyi Biotec.

### Flow cytometric phenotyping.

PBMCs were stained with a viability dye (LIVE/DEAD Fixable Aqua dead cell stain, Thermo Fisher Scientific) for 20 minutes at room temperature (RT). After washing twice with PBS, cells were incubated with anti-CXCR3–BV650 (G025H7, BioLegend) for 30 minutes at 37°C. Cells were then washed with staining buffer and stained with anti-CD57–PerCP-Cy5.5 (HNK-1, BioLegend), anti-CD56–FITC (B159, Becton Dickinson), anti-CD3–PE-Cy7 (SK7, Becton Dickinson), anti-NKp30–PE-CF594 (P30-1, BioLegend), anti-NKG2C–PE (134591, R&D Systems), anti-NKG2D–APC-Cy7 (1D11, BioLegend), anti-CD4–AF700 (RPA-T4, Becton Dickinson), anti-NKG2A–APC (Z199, Beckman Coulter), anti-CD16–BV786 (3G8, Becton Dickinson), anti-CD158b–BV605 (CH-L, Becton Dickinson), and anti-KLRG1–BV421 (14C2A07, BioLegend) for 20 minutes at RT. After washing, cells were then fixed with 1% paraformaldehyde (PFA) and acquired on an LSR Fortessa flow cytometer (Becton Dickinson). Data were analyzed using OMIQ software from Dotmatics (https://www.dotmatics.com/). Gating was performed according to the different FMO controls.

### NK cell functional activity assays.

NK cell activation and natural cytotoxicity was measured by the detection of CD107a and IFN-γ by flow cytometry in unstimulated NK cells and after coculture with K562 cells or primed with IL-15. Briefly, NK cells were isolated from cryopreserved PBMCs using a commercial kit (MagniSort Human NK cell Enrichment Kit). Then, NK and K562 cells were mixed at 1:1 ratio and cultured in R10 medium in the presence or absence of 25 ng/mL IL-15 (Miltenyi Biotec) for 4.5 hours in U-bottom 96-well plates at 37°C and 5% CO_2_. CD107a-PE-Cy5 (H4A3; Becton Dickinson), BD GolgiPlug Protein Transport Inhibitor (Becton Dickinson), and BD GolgiStop Protein Transport Inhibitor containing monensin (Becton Dickinson) were also added to each well at the recommended concentrations. Cells were then washed and stained with a viability dye (LIVE/DEAD Fixable Aqua dead cell stain). Subsequently, cells were stained with anti-CD56–FITC (B159, Becton Dickinson) and anti-CD3–PE-Cy7 (SK7, Becton Dickinson) antibodies for 20 minutes at RT. Cells were then fixed and permeabilized with Fixation/Permeabilization Solution (Becton Dickinson) for 20 minutes at 4°C, washed with BD Perm/Wash buffer, and stained with anti-IFN-γ–AF700 (B27, Life Technologies) for 30 minutes at RT. After washing, cells were fixed with 1% PFA and acquired on an LSR Fortessa flow cytometer. Data were analyzed using OMIQ software from Dotmatics.

### ADCC assays.

ADCC was measured as described by others, with some modifications (88). Briefly, ACH-2 cells were plated in round-bottom 96-well plates and incubated for 15 minutes with plasma (1:1000 dilution) from a viremic HIV^+^ PWH containing a mix of antibodies targeting different HIV epitopes. The same plasma, which does not contain neutralizing antibodies, was used for all ADCC experiments performed in this study. NK cells were isolated from PBMCs using magnetic beads (MagniSort Human NK cell Enrichment Kit) and added at 1:10 target/effector ratio. Plates were centrifuged at 400*g* for 3 minutes and incubated for 4 hours at 37°C and 5% CO_2_. After the coculture, cells were washed and stained with a viability dye (LIVE/DEAD Fixable Violet dead cell stain). Then, cells were stained with anti-CD56–FITC (B159, Becton Dickinson) and anti-CD3–PE-Cy7 (SK7, Becton Dickinson) for 20 minutes at RT. Cells were then fixed and permeabilized with Fixation/Permeabilization solution (Becton Dickinson) for 20 minutes at 4°C, washed with BD Perm/Wash buffer, and stained with anti-p24–PE (KC57-RD1, Beckman Coulter) for 30 minutes at 4°C and 30 minutes at RT. After washing, cells were fixed with 2% PFA and acquired on an LSR Fortessa flow cytometer. Data were analyzed using Flowjo v10 software.

### FACS of selected NK cell subsets from ECs.

For cell sorting, NK cells from ECs were isolated from PBMCs using a commercial kit (MagniSort Human NK cell Enrichment Kit). After enrichment, NK cells were stained with a viability dye (LIVE/DEAD Fixable Aqua Dead Cell stain) for 20 minutes at RT in the dark. Subsequently, cells were washed with 1× PBS and stained with anti-CXCR3–BV650 (G025H7, BioLegend) for 30 minutes at 37°C. Then, cells were washed with staining buffer and stained with anti-CD57–PerCP-Cy5.5 (HNK-1, BioLegend), anti-CD56–FITC (B159, Becton Dickinson), anti-CD3–PE-Cy7 (SK7, Becton Dickinson), anti-NKG2C–PE (134591, R&D Systems), anti-CD4–AF700 (RPA-T4, Becton Dickinson), anti-NKG2A–APC (Z199, Beckman Coulter), and anti-CD16–BV786 (3G8, Becton Dickinson) for 20 minutes at RT. Cells were then washed with staining buffer and immediately sorted using the Cytek Aurora Cell Sorter. We sorted live NK cell populations (CD3^–^CD4^–^CD56^+^). Then, CD16^+^ cells derived from CD56^+^ NK cells were classified based on the expression of NKG2A, NKG2C, CD57, and CXCR3. NK cell classification included NKA (NKG2A^+^NKG2C^+^CD57^+^CXCR3^+^), NKB (NKG2A^–^NKG2C^+^CD57^+^CXCR3^+^), NKC (NKG2A^+^NKG2C^–^CD57^+^CXCR3^+^), and NKD (NKG2A^–^NKG2C^–^CD57^+^CXCR3^+^). CD16^–^ NK cells were designated as NKE. Cell numbers obtained after sorting of selected NK cell populations are shown in Supplemental Table 2.

### Dimensionality reduction analysis.

Dimensionality reduction analysis (optimized t-distributed stochastic neighbor embedding, opt-SNE) of flow cytometry data was performed in OMIQ. All events within pregated CD56^+^ NK cells were concatenated for each group per group and culture condition and analyzed. Clustering analysis was performed via Flow-Self Organizing Maps (FlowSOM) artificial intelligence algorithm on equal samples of randomly selected 7 × 10^5^ cells from each group. For phenotypic characterization, clustering was performed based on the expression of the markers CD57, NKp30, NKG2C, NKG2D, NKG2A, CD16, CXCR3, CD158b, and KLRG1. Marker expression was normalized and represented as arcsinh-transformed medians within heatmaps generated via opt-SNE analysis (89).

### Gene expression analysis by single-cell RNA-seq.

NK cell samples enriched in specific receptors were obtained through FACS, as described in the previous section. A total of 529,800 cells were isolated from the NKA subset, 170,000 from the NKB subset, and 370,000 from the NKC subset. Subsequently, RNA-seq libraries were prepared at the Josep Carreras Institute using the Chromium single-cell RNA sequencing (scRNAseq) 3′ protocol and were subjected to sequencing on the NovaSeq PE150 platform at Novogene. The Cell Ranger pipeline was employed for read alignment to the human genome, cell calling, and generation of the raw counts matrix. Quality controls were conducted using FastQC v0.11.9 to ensure data was appropriate for downstream analyses. Based on the quality control outcomes, a conservative filtering approach was applied, considering the number of features per cell and mitochondrial/ribosomal counts, to exclude cells of low quality. Subsequent to alignment and quantification of reads to annotated mRNAs using the Cell Ranger count tool, data normalization was executed using SCTransform. Dimensionality reduction was achieved through Principal Component Analysis (PCA) based on the top 3000 most variable features, and the first 30 principal components (PCs) were retained for subsequent analyses. Cell clustering was performed using an SNN modularity optimization–based clustering algorithm. Visualization of cell distribution based on sample type was accomplished through UMAP. Cluster marker identification and differential expression analyses were conducted using the FindMarkers function from the Seurat package. The assessment of biological significance was conducted through enrichment analysis utilizing the GO Biological Process category database. Additionally, differentiation trajectory analysis was carried out using the Slingshot package. Bioinformatics analysis was carried out in the Statistics and Bioinformatics Unit (UEB) at Vall d’Hebron Research Institute (VHIR).

### Statistics.

Number of individuals per group are described in the figure legends. All figure panels include data sets obtained from individual samples. All graphing and statistical analyses were performed using GraphPad Prism 8.3. Significance of differences between all experimental groups was determined using a 1-way or 2-way ANOVA followed by Dunn’s multiple-comparison test or Tukey’s multiple-comparison test, and are reported within each figure legend. A *P* value of less than 0.05 was considered significant: *P < 0.05, **P < 0.01, ***P < 0.001, ****P < 0.0001. Tables were prepared using Microsoft Word and Excel.

### Study approval.

Written informed consent for sample collection and the use of medical record information was obtained from all participants included in the study. This study was approved by the corresponding Ethical Committees of the Vall d’Hebron University Hospital (VHUH), Barcelona, Spain (Institutional Review Board numbers PR(AG)270/2015, PR(AG)192/2018, and PR(AG)476-2018).

### Data availability.

All data associated with this study are provided within the paper or the supplemental information, and raw data are included in the Supporting Data Values file. Sequencing data are available in the NCBI GEO under accession number GSE273423.

## Author contributions

NSG and MJB designed, directed, and interpreted experiments. NSG, AGC, AAG and MG designed and performed experiments, analyzed the data, and interpreted experiments. NR, JMB, ERM, AC, JB, JN, PS, and VF were responsible for the recruitment, specimen handling and storage, and related clinical data collection. NSG and MJB wrote the initial manuscript, and all of us contributed to the editing of the manuscript.

## Supplementary Material

Supplemental data

Supporting data values

## Version 1 09/17/2024

In-Press Preview

## Version 2 10/22/2024

Electronic publication

## References

[1] Cao Y (1995). Virologic and immunologic characterization of long-term survivors of human immunodeficiency virus type 1 infection. N Engl J Med.

[2] Pantaleo G (1995). Studies in subjects with long-term nonprogressive human immunodeficiency virus infection. N Engl J Med.

[3] O’Connell KA (2010). Control of HIV-1 in elite suppressors despite ongoing replication and evolution in plasma virus. J Virol.

[4] Deeks SG, Walker BD (2007). Human immunodeficiency virus controllers: mechanisms of durable virus control in the absence of antiretroviral therapy. Immunity.

[5] Jiang C (2020). Distinct viral reservoirs in individuals with spontaneous control of HIV-1. Nature.

[6] Autran B (2011). Elite controllers as a model of functional cure. Curr Opin HIV AIDS.

[7] Mens H (2010). HIV-1 continues to replicate and evolve in patients with natural control of HIV infection. J Virol.

[8] Miura T (2010). Impaired replication capacity of acute/early viruses in persons who become HIV controllers. J Virol.

[9] Casado C (2020). Permanent control of HIV-1 pathogenesis in exceptional elite controllers: a model of spontaneous cure. Sci Rep.

[10] Fellay J (2007). A whole-genome association study of major determinants for host control of HIV-1. Science.

[11] Navarrete-Muñoz MA (2020). Elite controllers: A heterogeneous group of HIV-infected patients. Virulence.

[12] Pernas M (2018). Factors leading to the loss of natural elite control of HIV-1 infection. J Virol.

[13] Grabar S (2009). Prevalence and comparative characteristics of long-term nonprogressors and HIV controller patients in the French Hospital Database on HIV. AIDS.

[14] Bailey JR (2006). Maintenance of viral suppression in HIV-1-infected HLA-B*57+ elite suppressors despite CTL escape mutations. J Exp Med.

[15] Altfeld M (2003). Influence of HLA-B57 on clinical presentation and viral control during acute HIV-1 infection. AIDS.

[16] Walker BD, Yu XG (2013). Unravelling the mechanisms of durable control of HIV-1. Nat Rev Immunol.

[17] May ME (2020). Combined effects of HLA-B*57/5801 elite suppressor CD8+ T cells and NK cells on HIV-1 replication. Front Cell Infect Microbiol.

[18] Emu B (2008). HLA class I-restricted T-cell responses may contribute to the control of human immunodeficiency virus infection, but such responses are not always necessary for long-term virus control. J Virol.

[19] Jansen CA (2005). High responsiveness of HLA-B57-restricted Gag-specific CD8+ T cells in vitro may contribute to the protective effect of HLA-B57 in HIV-infection. Eur J Immunol.

[20] Collins DR (2021). Functional impairment of HIV-specific CD8^+^ T cells precedes aborted spontaneous control of viremia. Immunity.

[21] Shi Y (2022). The role of innate immunity in natural elite controllers of HIV-1 infection. Front Immunol.

[22] Sugawara S (2022). Learning to be elite: lessons from HIV-1 controllers and animal models on trained innate immunity and virus suppression. Front Immunol.

[23] Vivier E (2008). Functions of natural killer cells. Nat Immunol.

[24] Lanier LL (2008). Up on the tightrope: natural killer cell activation and inhibition. Nat Immunol.

[25] Mikulak J (2017). Natural killer cells in HIV-1 infection and therapy. AIDS.

[26] Bernard NF (2022). Natural killer cells in antibody independent and antibody dependent HIV control. Front Immunol.

[27] Martin MP (2007). Innate partnership of HLA-B and KIR3DL1 subtypes against HIV-1. Nat Genet.

[28] Martin MP (2018). Killer cell immunoglobulin-like receptor 3DL1 variation modifies HLA-B*57 protection against HIV-1. J Clin Invest.

[29] Gumá M (2004). Imprint of human cytomegalovirus infection on the NK cell receptor repertoire. Blood.

[30] Rückert T (2022). Clonal expansion and epigenetic inheritance of long-lasting NK cell memory. Nat Immunol.

[31] Kant S (2020). Polyfunctional Fc dependent activity of antibodies to native trimeric envelope in HIV elite controllers. Front Immunol.

[32] O’Connor GM (2007). Natural Killer cells from long-term non-progressor HIV patients are characterized by altered phenotype and function. Clin Immunol.

[33] Zhang Z (2021). Changes in NK cell subsets and receptor expressions in HIV-1 infected chronic patients and HIV controllers. Front Immunol.

[34] Pohlmeyer CW (2019). Identification of NK cell subpopulations that differentiate HIV-infected subject cohorts with diverse levels of virus control. J Virol.

[35] Study IHC (2010). The major genetic determinants of HIV-1 control affect HLA class I peptide presentation. Science.

[36] Barnes S (2020). Deciphering the immunological phenomenon of adaptive natural killer (NK) cells and cytomegalovirus (CMV). Int J Mol Sci.

[37] Lopez-Vergès S (2010). CD57 defines a functionally distinct population of mature NK cells in the human CD56dimCD16+ NK-cell subset. Blood.

[38] Gondois-Rey F (2017). A mature NK profile at the time of HIV primary infection is associated with an early response to cART. Front Immunol.

[39] Muntasell A (2013). Adaptive reconfiguration of the human NK-cell compartment in response to cytomegalovirus: a different perspective of the host-pathogen interaction. Eur J Immunol.

[40] Hearps AC (2024). Adaptive NK cells rapidly expand during acute HIV infection and persist despite early initiation of antiretroviral therapy. J Immunol.

[41] Angelo LS (2015). Practical NK cell phenotyping and variability in healthy adults. Immunol Res.

[42] Kaulfuss M (2023). The NK cell checkpoint NKG2A maintains expansion capacity of human NK cells. Sci Rep.

[43] Cooper MA, Fehniger TA (2001). The biology of human natural killer-cell subsets. Trends Immunol.

[44] Jacobs R (2001). CD56bright cells differ in their KIR repertoire and cytotoxic features from CD56dim NK cells. Eur J Immunol.

[45] Poli A (2009). CD56bright natural killer (NK) cells: an important NK cell subset. Immunology.

[46] https://qubeshub.org/resources/fastqc.

[47] Butler A (2018). Integrating single-cell transcriptomic data across different conditions, technologies, and species. Nat Biotechnol.

[48] Ewels P (2016). MultiQC: summarize analysis results for multiple tools and samples in a single report. Bioinformatics.

[49] Yang C (2019). Heterogeneity of human bone marrow and blood natural killer cells defined by single-cell transcriptome. Nat Commun.

[50] Kim SH (2023). Enhancement of the anticancer ability of natural killer cells through allogeneic mitochondrial transfer. Cancers (Basel).

[51] Cherrier DE (2018). Innate lymphoid cell development: a T cell perspective. Immunity.

[52] Duhan V (2019). NK cell-intrinsic FcεRIγ limits CD8+ T-cell expansion and thereby turns an acute into a chronic viral infection. PLoS Pathog.

[53] Eisele E, Siliciano RF (2012). Redefining the viral reservoirs that prevent HIV-1 eradication. Immunity.

[54] Altfeld M (2006). HLA alleles associated with delayed progression to AIDS contribute strongly to the initial CD8(+) T cell response against HIV-1. PLoS Med.

[55] Sáez-Cirión A (2007). HIV controllers exhibit potent CD8 T cell capacity to suppress HIV infection ex vivo and peculiar cytotoxic T lymphocyte activation phenotype. Proc Natl Acad Sci U S A.

[56] Mela CM (2005). Switch from inhibitory to activating NKG2 receptor expression in HIV-1 infection: lack of reversion with highly active antiretroviral therapy. AIDS.

[57] Pace M (2022). Impact of antiretroviral therapy in primary HIV infection on natural killer cell function and the association with viral rebound and HIV DNA following treatment interruption. Front Immunol.

[58] Nielsen CM (2013). Functional significance of CD57 expression on human NK cells and relevance to disease. Front Immunol.

[59] Tomescu C (2021). Identification of the predominant human NK cell effector subset mediating ADCC against HIV-infected targets coated with BNAbs or plasma from PLWH. Eur J Immunol.

[60] Climent N (2023). Immunological and virological findings in a patient with exceptional post-treatment control: a case report. Lancet HIV.

[61] Siemaszko J (2023). Activating NKG2C receptor: functional characteristics and current strategies in clinical applications. Arch Immunol Ther Exp (Warsz).

[62] Kusumi M (2006). Expression patterns of lectin-like natural killer receptors, inhibitory CD94/NKG2A, and activating CD94/NKG2C on decidual CD56bright natural killer cells differ from those on peripheral CD56dim natural killer cells. J Reprod Immunol.

[63] Béziat V (2011). Human NKG2A overrides NKG2C effector functions to prevent autoreactivity of NK cells. Blood.

[64] Davis ZB (2016). A conserved HIV-1-derived peptide presented by HLA-E renders infected T-cells highly susceptible to attack by NKG2A/CD94-bearing natural killer cells. PLoS Pathog.

[65] Ali A (2021). Natural killer cell immunosuppressive function requires CXCR3-dependent redistribution within lymphoid tissues. J Clin Invest.

[66] Hoehl S (2020). Thirty years of CMV seroprevalence-a longitudinal analysis in a German university hospital. Eur J Clin Microbiol Infect Dis.

[67] Grønborg HL (2017). Review of cytomegalovirus coinfection in HIV-infected individuals in Africa. Rev Med Virol.

[68] Albasanz-Puig A (2021). Low frequency of cytomegalovirus (CMV) disease despite high prevalence of CMV viraemia in patients with advanced HIV infection: a clinical and immunological 48-week follow-up study. HIV Med.

[69] Haynes BF (2012). Immune-correlates analysis of an HIV-1 vaccine efficacy trial. N Engl J Med.

[70] Lichtfuss GF (2012). Virologically suppressed HIV patients show activation of NK cells and persistent innate immune activation. J Immunol.

[71] Ackerman ME (2016). Polyfunctional HIV-specific antibody responses are associated with spontaneous HIV control. PLoS Pathog.

[72] Lambotte O (2009). Heterogeneous neutralizing antibody and antibody-dependent cell cytotoxicity responses in HIV-1 elite controllers. AIDS.

[73] Wren LH (2013). Specific antibody-dependent cellular cytotoxicity responses associated with slow progression of HIV infection. Immunology.

[74] Kammers K (2021). HIV antibody profiles in HIV controllers and persons with treatment-induced viral suppression. Front Immunol.

[75] Astorga-Gamaza A (2022). Identification of HIV-reservoir cells with reduced susceptibility to antibody-dependent immune response. Elife.

[76] Vieillard V (2010). Specific phenotypic and functional features of natural killer cells from HIV-infected long-term nonprogressors and HIV controllers. J Acquir Immune Defic Syndr.

[77] Kuri-Cervantes L (2014). Activation of NK cells is associated with HIV-1 disease progression. J Leukoc Biol.

[78] Naranbhai V (2013). Changes in Natural Killer cell activation and function during primary HIV-1 Infection. PLoS One.

[79] Alter G (2007). Single-stranded RNA derived from HIV-1 serves as a potent activator of NK cells. J Immunol.

[80] Fox JC (2015). Structural and agonist properties of XCL2, the other member of the C-chemokine subfamily. Cytokine.

[81] Guzzo C (2013). The CD8-derived chemokine XCL1/lymphotactin is a conformation-dependent, broad-spectrum inhibitor of HIV-1. PLoS Pathog.

[82] Rasool ST (2008). Increased level of IL-32 during human immunodeficiency virus infection suppresses HIV replication. Immunol Lett.

[83] Ribeiro-Dias F (2017). Interleukin 32: a novel player in the control of infectious diseases. J Leukoc Biol.

[84] Flórez-Álvarez L (2018). NK cells in HIV-1 infection: from basic science to vaccine strategies. Front Immunol.

[85] Cantero-Pérez J (2019). Resident memory T cells are a cellular reservoir for HIV in the cervical mucosa. Nat Commun.

[86] Jurczyszak D (2022). ISG15 deficiency restricts HIV-1 infection. PLoS Pathog.

[87] Mathieu NA (2021). HERC5 and the ISGylation pathway: critical modulators of the antiviral immune response. Viruses.

[88] Davis-Gardner ME (2017). eCD4-Ig promotes ADCC activity of sera from HIV-1-infected patients. PLoS Pathog.

[89] Braanker H den (2021). How to prepare spectral flow cytometry datasets for high dimensional data analysis: a practical workflow. Front Immunol.

