# Supplementary material for: NKG2C and NKG2A coexpression defines a highly functional antiviral NK population in spontaneous HIV control
Source: JCI Insight. 2024 Sep 17;9(20):e182660. doi: 10.1172/jci.insight.182660 (PMC11529982; doi:10.1172/jci.insight.182660)
Supplement: Supplemental data [file jciinsight-9-182660-s250.pdf]

Supplementary Data

NKG2C and NKG2A Co-expression Defines a Highly Functional Antiviral NK Population in Spontaneous HIV Control

Sanchez-Gaona et al.

Supplementary Figure 1

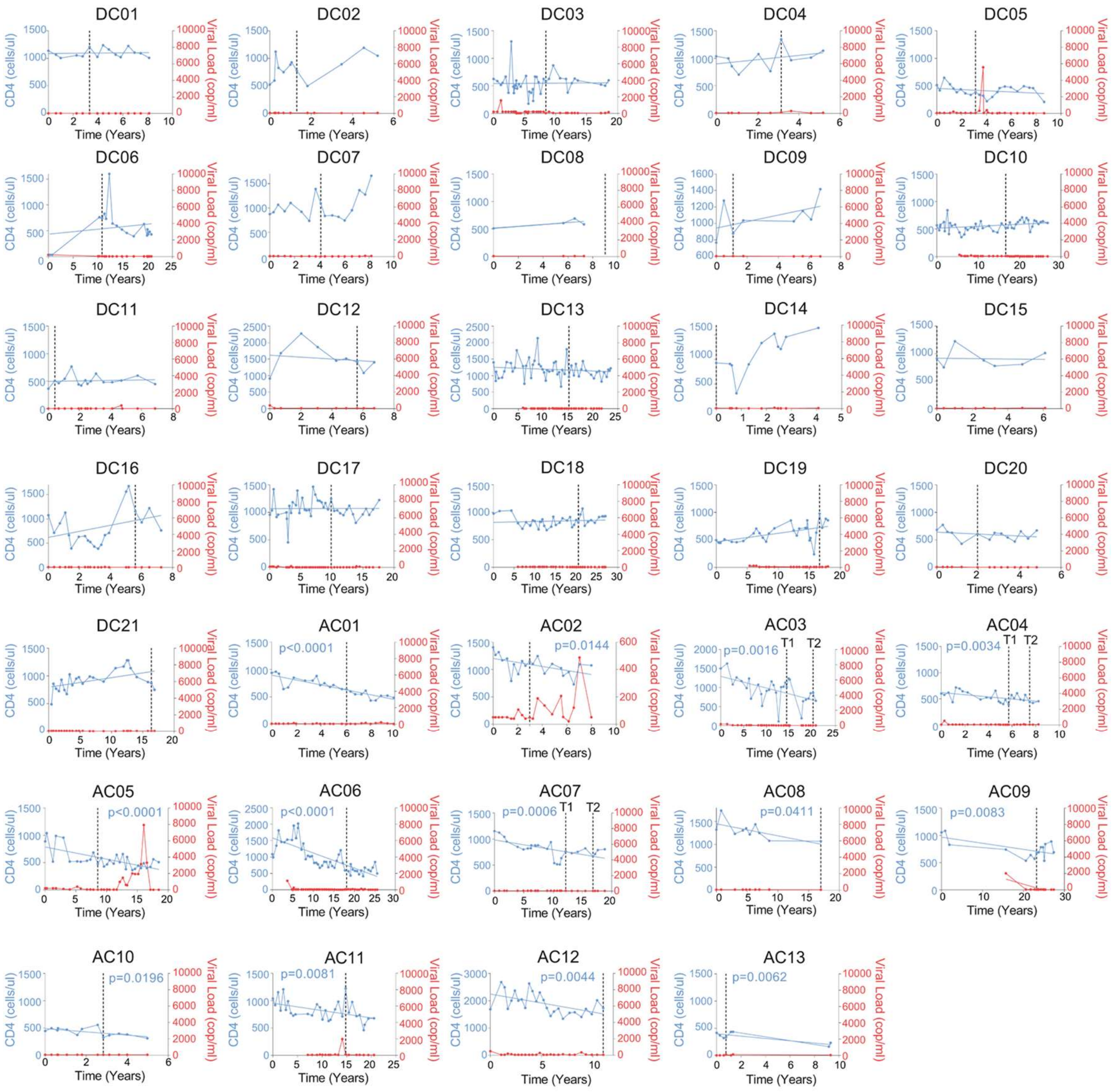

Supplementary Figure 1. Viral and immune dynamics in study participants. Viral loads (right axes) and CD4 counts (left axes) over time (x-axis) for subjects with durable (DC01 - DC21) and aborted control (AC01 - AC013). Timepoints of study sample collection are represented with dashed lines.

Supplementary Figure 2

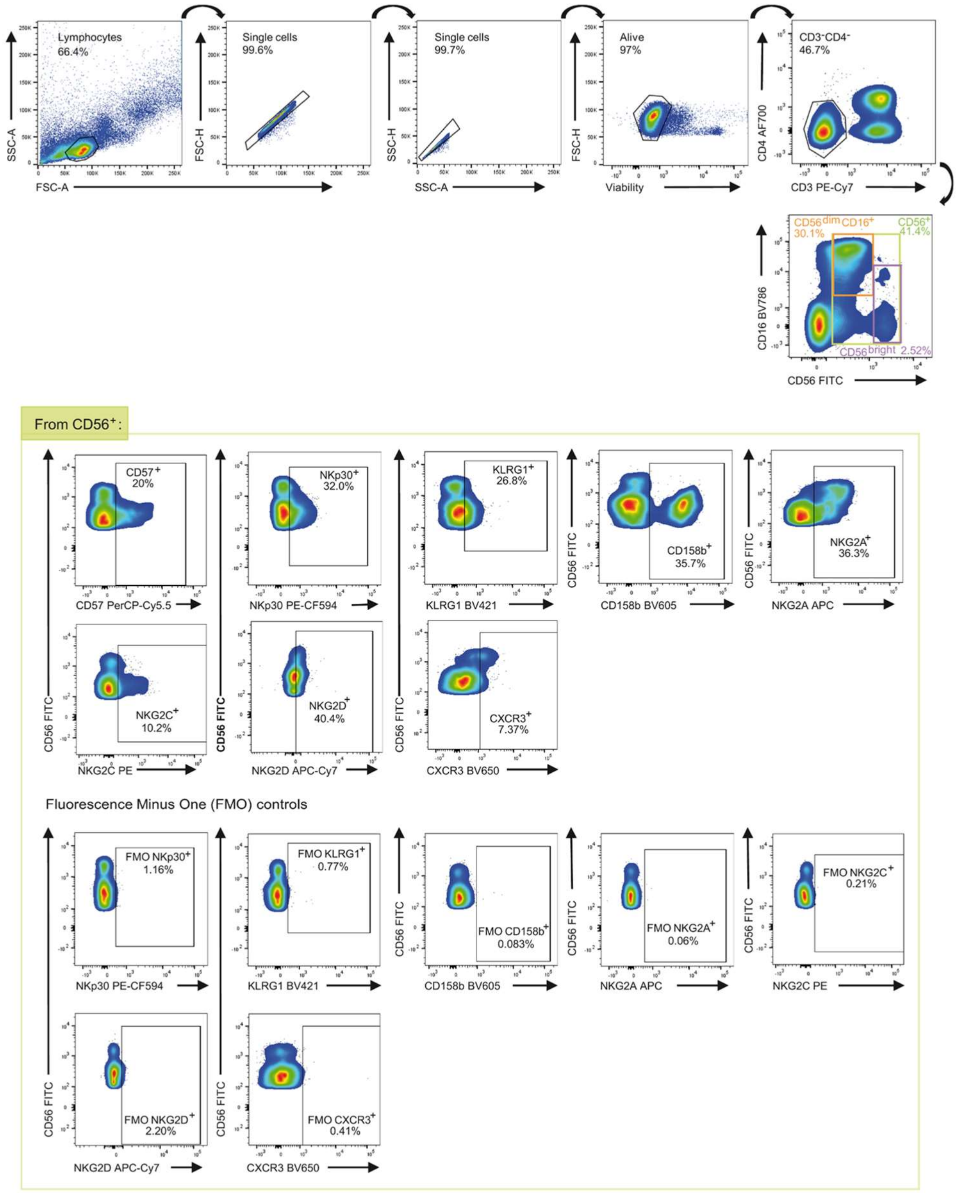

Supplementary Figure 2. Gating strategy used for the quantification of the different NK cell receptors. General gating strategy used to identify NK cell populations based on CD56 and CD16 expression from PBMC samples for phenotype and functional assays. Briefly, lymphocytes were selected based on FSC and SSC parameters, followed by doublets and dead cells exclusion. From CD3$^-$ CD4$^-$ cells, NK cells (defined as CD3$^-$CD56$^+$) were gated; and within NK cells, three subsets were identified in all groups: CD56$^+$, CD56^{dim}CD16$^+$ and CD56^{bright}. FMOs are shown for markers with continuous expression. *FSC-H, Forward Scatter-Height; FSC-A, Forward Scatter-Area; SSC-A, Side Scatter-Area.*

Supplementary Figure 3

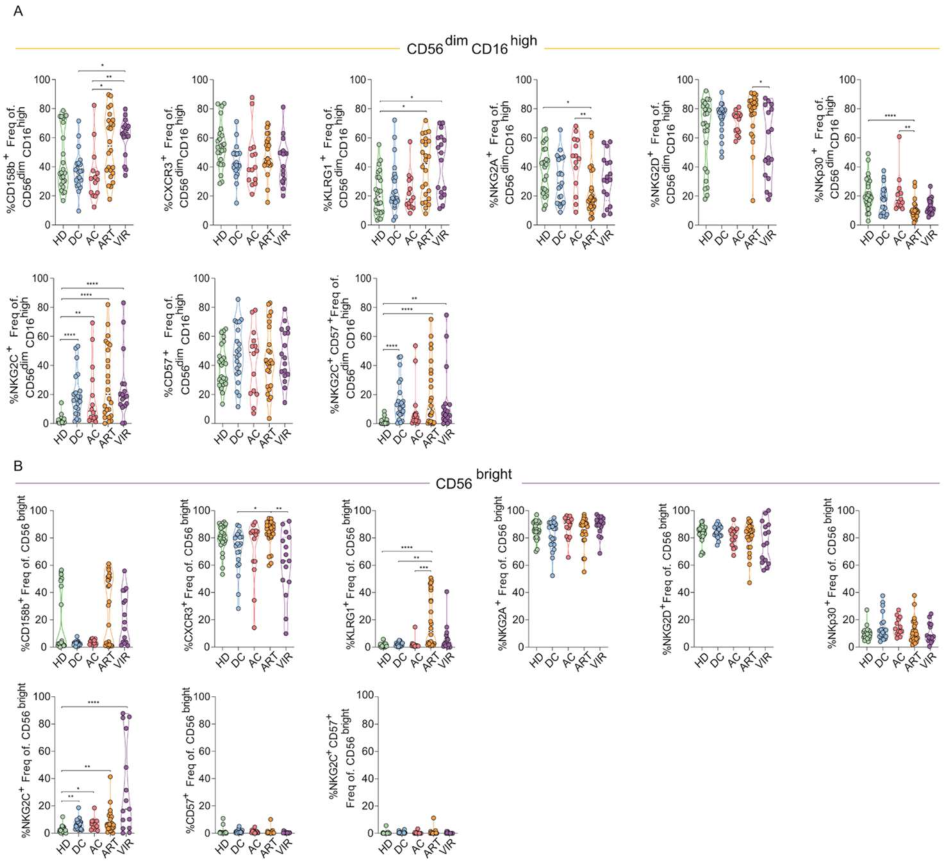

Supplementary Figure 3. **Phenotypic characterization of NK cell subsets**. **A**) Violin plots depicting the expression of distinct phenotypic NK cell markers in $CD56^{dim}CD16^{high}$ NK cells (left to right: CD158b, CXCR3, KLRG1, NKG2A, NKG2D, NKp30, NKG2C, CD57, and the frequency of double-positive $NKG2C^{+}CD57^{+}$ NK cells). **B**) Violin plots showing the frequency of (left to right): $CD158b^{+}$, $CXCR3^{+}$, $KLRG1^{+}$, $NKG2A^{+}$, $NKG2D^{+}$, $NKp30^{+}$, $NKG2C^{+}$, and $CD57^{+}$ $CD56^{bright}$ NK cells. Graphs represent medians and ranges. Statistical comparisons were performed using the Kruskal-Wallis test. *p<0.05; **p<0.01; ***p<0.001; ****p<0.0001.

Supplementary Figure 4

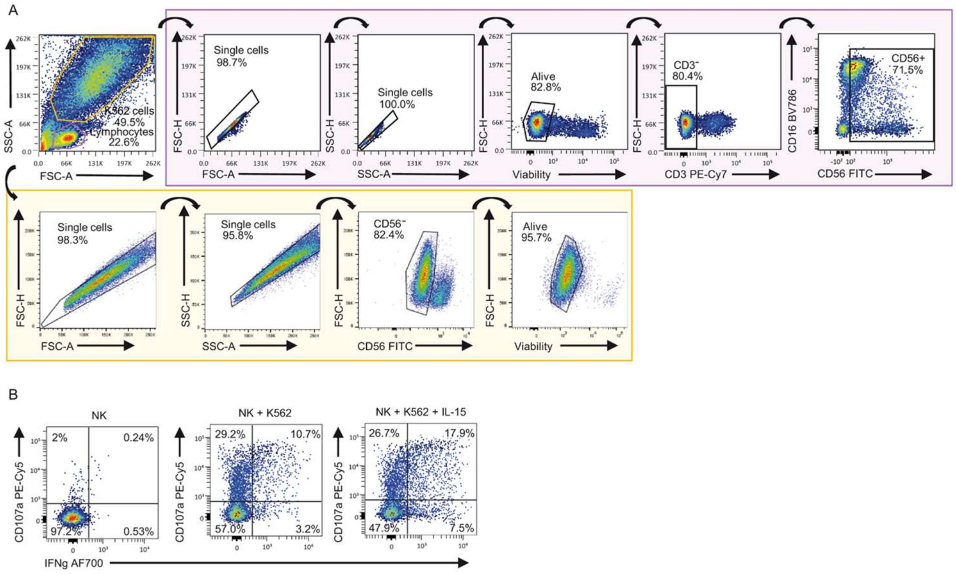

Supplementary Figure 4. Gating strategy for NK cell activation assays. A) General gating strategy used to identify NK cells and K562 cells in NK cell activation and cytotoxicity assays. For NK cells (labelled in purple), lymphocytes were selected based on FSC and SSC parameters, followed by doublets and dead cells exclusion. From $CD3^-$ cells, $CD56^+$ NK cells were selected. Similarly, K562 cells (labelled in yellow) were selected based on FSC and SSC parameters, followed the exclusion of doublet cells. Subsequently, $CD56^-$ cells live cells were selected. **B**) Representative flow cytometry plots illustrating the expression of $IFN\gamma^+CD107a^+$ in $CD56^+$ NK cells following stimulation with K562 and IL-15 are shown. *FSC-H, Forward Scatter-Height; FSC-A, Forward Scatter-Area; SSC-A, Side Scatter-Area.*

Supplementary Figure 5

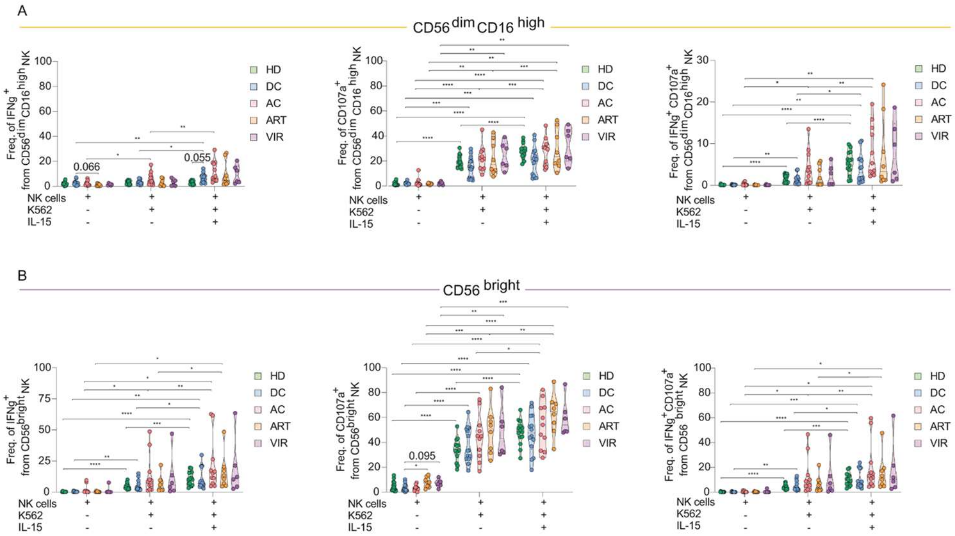

Supplementary Figure 5. Functional profile of NK cell subsets. Violin plots depicting the frequency of IFNɣ$^{+}$, CD107a^{+}, and IFNɣ$^{+}$CD107a^{+} in **A**) CD56^{dim}CD16^{high} NK cells and **B**) CD56^{bright} NK cells upon stimulation. Statistical comparisons were performed using RM two-way ANOVA followed by Tukey's multiple comparisons test. *$p<0.05$; **$p<0.01$; ***$p<0.001$; ****$p<0.0001$.

Supplementary Figure 6

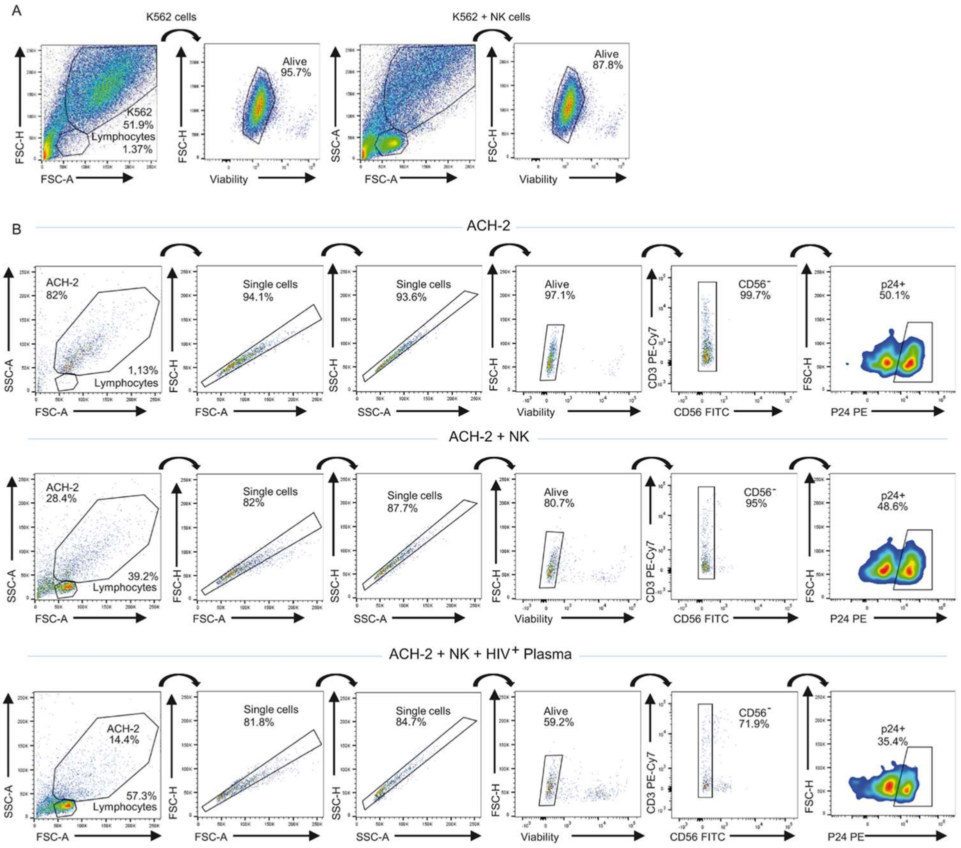

Supplementary Figure 6. **Gating strategy for functional assays. A**) General gating strategy used to assess natural cytotoxicity. The viability reduction of K562 cells in co-culture with NK cells was quantified as the percentage difference between the viability of K562 cells alone and K562 cells co-cultured with NK cells. **B**) Representative flow cytometry plots showing the gating strategy used to evaluate ADCC responses. Primary NK cells were isolated and co-cultured with the p24-expressing ACH-2 cell line, with and without the addition of HIV$^+$ plasma for 4h. ACH-2 cells were gated based on SSC-A vs FSC-A plot. After doublet cell exclusion, live cells were identified based on the lack of expression of LIVE/DEAD Fixable AQUA viability cell stain. Then, p24$^+$ expression was analyzed in CD56$^-$ cells. ACH-2 cells alone, co-cultured with NK cells and with HIV$^+$ plasma are depicted below. ADCC was calculated as the fraction of cells that disappeared within the target population after the addition of HIV$^+$ plasma in comparison to the control condition with targets and NK cells, but lacking HIV$^+$ plasma. *FSC-H, Forward Scatter-Height; FSC-A, Forward Scatter-Area; SSC-A, Side Scatter-Area.*

Supplementary Figure 7

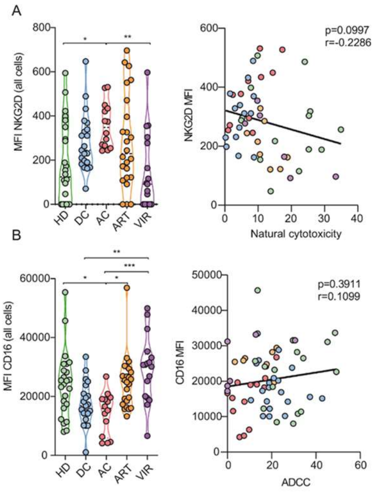

Supplementary Figure 7. Expression levels of activating NK cell markers and their association with functional responses. A) Mean Fluorescence Intensity (MFI) of NKG2D on total CD56^{+} NK cells across study groups (left panel) and Spearman correlation illustrating its association with natural cytotoxic responses (right panel). **B**) MFI of CD16 on total CD56^{+} NK cells across study groups (left panel) and Spearman correlation showing its association with ADCC responses (right panel).

Supplementary Figure 8

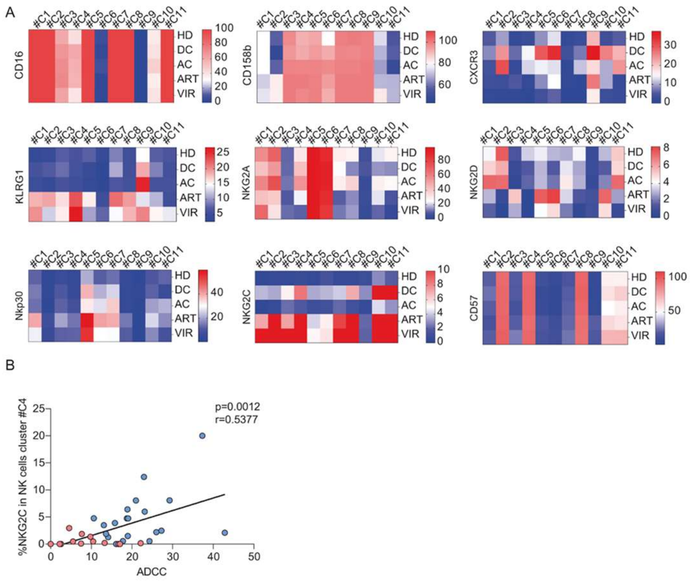

Supplementary Figure 8. NK cell receptor expression within NK cell clusters. A) Heatmaps depicting the frequency of expression of the selected phenotypic NK cell markers within the cell clusters identified in Figure 2A, categorized by study group. Markers include CD16, CD158b, CXCR3, KLRG1, NKG2A, NKG2D, NKp30, NKG2C, and CD57. **B**) Spearman correlation illustrating the association between NKG2C expression in NK cells from cluster #C4 and ADCC responses in DC and AC groups (DC in blue, AC in red).

Supplementary Figure 9

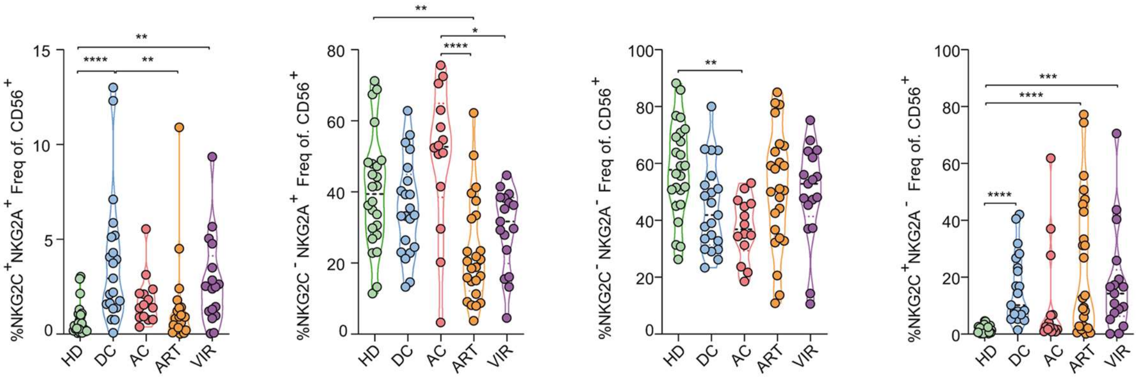

Supplementary Figure 9. **Frequency of NK cell populations based on NKG2C and NKG2A expression**. Violin plots illustrating the frequency of distinct NK cell populations characterized by NKG2C and NKG2A expression, specifically (left to right): NKG2C^{+}NKG2A^{+}, NKG2C^{-}NKG2A^{+}, NKG2C^{-}NKG2A^{-}, and NKG2C^{+}NKG2A^{-} total CD56^{+} NK cells. Graphs represent medians and ranges. Statistical comparisons were conducted using the Kruskal-Wallis test, with significance levels indicated as follows: *p<0.05; **p<0.01; ***p<0.001; ****p<0.0001.

Supplementary Figure 10

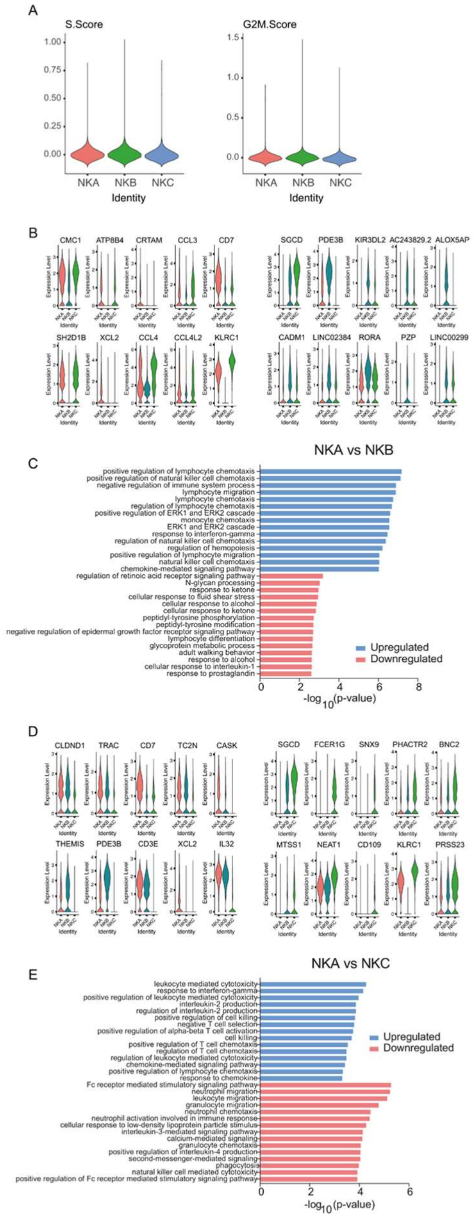

Supplementary Figure 10. Influence of cell cycle dynamics and single-cell differential gene expression analysis between NK cell populations. A) Violin plots illustrating the fraction of cells in different phases of the cell cycle (S left, G2M right) across NK cell samples. Cells are colored by sample classification (NKA red, NKB green, and NKC blue). **B**) Top ten genes exhibiting upregulation (left) and downregulation (right) in NKA cells compared to NKB. **C**) Overrepresentation analysis (ORA) of the gene ontology biological process (GO-BP) analysis showing the list of biological processes upregulated (blue) or downregulated (red) in NKA vs NKB. **D**) Top ten genes upregulated (left) and downregulated (right) in NKA cells compared to NKC. **E**) Overrepresentation analysis (ORA) of the gene ontology biological process (GO-BP) analysis showing the list of biological processes upregulated (blue) or downregulated (red) in NKA vs NKC based on the list of genes upregulated and downregulated in each comparison with an adjusted p-value below 0.05 and log2FoldChange above 0.5 (upregulated) or below -0.5 (downregulated).

Table S1. Clinical data of PWH included in the study.

Subject characteristics		DC (n=21)	AC (n=13)	ART (n=24)	VIR (n=18)	p value between groups(test)
Sex (n, %)	Male	11 (55)	7 (53)	15 (65.2)	14 (77.7)	0.35 (Fisher's exact)
	Female	9 (45)	6 (46)	8 (34.8)	4 (22.2)	
Age (years; median, IQR)		55 (47 -59)	58 (51-61)	44 (36-52)	39 (27-46)	**<0.0001** (Kruskal-Wallis test with Dunn's post-test)
Route of transmission (n, %)	Heterosexual	6 (30)	7 (53.8)	3 (37.5)	2 (28.6)	**<0.0001** (Fisher's exact)
	MSM	4 (20)	1 (7.6)	4 (50)	5 (71.4)	
	Intravenous drug use	10 (50)	3 (23)	1 (12.5)	-	
	Other/unknown	-	2 (15.8)	-	-	
Protective HLA-B alleles [B*14. B*27. B*52. B*57] (n, %)		13 (72.2)	9 (69.2)	-	-	>0.99 (Fisher's exact)
CD4+ T-cell count (cells/ul; median, IQR)		846 (590.3-1109)	673 (501.8-1090)	750 (510-860)	435 (237.5-715)	**0.0005** (Kruskal-Wallis test with Dunn's post-test)
HIV viral load (log10 copies/ml; median, IQR)		<1.69	<1.69	<1.69	4.75 (4.38-5.25)	**<0.0001** (Kruskal-Wallis test with Dunn's post-test)
Time since HIV diagnosis (months; median, IQR)		87 (55.8-184.8)	132 (70.80-214.8)	74.80 (46.90-153.7)	8 (0.4-82.2)	**0.006** (Kruskal-Wallis test with Dunn's post-test)
Time on ART with VL suppressed (months; median, IQR)		N/A	N/A	53 (45-72)	N/A	

DC: Durable Control, AC: Aborted Control, ART: ART-treated PWH, VIR: Viremic PWH, IQR: Interquartile Range, MSM: Men who have sex with men; N/A not available

Table S2. Cell numbers obtained after sorting of selected NK cell populations.

	Cell population				
Sample	***NKA***	***NKB***	***NKC***	***NKD***	***NKE***
DC1	*1,043*	*5391*	*9,339*	*7,391*	*23,976*
DC2	*1,751*	*88,040*	*5,279*	*9,436*	*86,222*
DC3	*1,275*	*3,910*	*5,630*	*3,140*	*187,785*
DC4	*1,352*	*26,599*	*22,236*	*51,191*	*257,153*
DC5	*1,033*	*2,906*	*29,916*	*32,298*	*92,687*

DC: Durable Control, NKA: $CD16^+NKG2A^+NKG2C^+$ $CD57^+CXCR3^+$ NK cells, NKB: CD16+, $NKG2A^+$ $NKG2C^-$ $CD57^+$ $CXCR3^+$ NK cells, NKC: CD16+ $NKG2A^-$ $NKG2C^+$ $CD57^+$ $CXCR3^+$ NK cells, NKD: CD16+, $NKG2A^-$ $NKG2C^-$ $CD57^+$ $CXCR3^+$ NK cells, NKE: CD16- NK cells.

Table S3. Differentially expressed genes in each cluster.

p-val	Avg_log2FC	Pct.1	Pct.2	P_val_adj	Cluster	gene
0	0.817808347	0.864	0.517	0	0	CD7
0	0.775222722	0.993	0.666	0	0	IL32
0	0.660553583	0.905	0.51	0	0	CD3E
0	0.466822812	1	1	0	0	MT-CYB
0	0.432707859	1	1	0	0	MT-CO1
0	0.40040603	1	1	0	0	CCL5
0	0.398049407	1	1	0	0	MT-ATP6
0	0.375945164	0.348	0.096	0	0	GOLGA8M
9.3318E-300	0.420869948	1	1	2.0949E-295	0	MT-ND2
4.4041E-297	0.434556085	0.999	0.997	9.8867E-293	0	MT-ND5
6.6557E-232	0.378199728	1	1	1.4941E-227	0	RPLP1
4.6266E-229	0.380241959	0.999	0.991	1.0386E-224	0	CD52
7.2756E-211	0.449718029	0.926	0.828	1.6333E-206	0	S100A10
1.9296E-203	0.460477722	0.921	0.827	4.3318E-199	0	HOPX
6.0427E-197	0.43411818	0.549	0.293	1.3565E-192	0	CASK
1.1308E-192	0.473530087	0.894	0.772	2.5385E-188	0	IFITM2
2.0933E-182	0.368429246	0.954	0.889	4.6992E-178	0	CD63
9.9805E-166	0.451925834	0.931	0.857	2.2405E-161	0	VIM
1.7646E-164	0.393350847	0.724	0.508	3.9613E-160	0	TRAC
2.36615E-85	0.403214858	0.719	0.596	5.31178E-81	0	LGALS1
0	1.633328618	0.999	0.88	0	1	CCL4
0	1.289329938	0.621	0.101	0	1	SNX9
0	1.220976009	0.998	0.649	0	1	KLRC1
0	1.132874697	0.915	0.483	0	1	SGCD
0	1.120477702	0.947	0.637	0	1	IGF2R
0	1.108214972	0.81	0.177	0	1	FCER1G
0	0.992366798	0.835	0.506	0	1	BNC2
0	0.988083512	0.94	0.665	0	1	ZFP36L1
0	0.985956975	0.868	0.489	0	1	CCL3

0	0.985469541	0.479	0.061	0	1	CD109
0	0.907454895	0.998	0.953	0	1	NEAT1
0	0.8798496	0.756	0.386	0	1	GAS7
0	0.868009861	0.731	0.288	0	1	FAM3C
0	0.864882042	0.699	0.315	0	1	GFOD1
0	0.856125723	0.937	0.708	0	1	SLA
0	0.824201961	0.899	0.634	0	1	PTPN12
0	0.794546607	0.958	0.762	0	1	ARID5B
0	0.787932632	0.642	0.204	0	1	NR4A2
0	0.750772754	0.999	0.973	0	1	LYST
6.7483E-210	0.788017608	0.505	0.257	1.5149E-205	1	BACH2
0	1.823706877	0.978	0.441	0	2	PDE3B
0	1.263094489	0.839	0.33	0	2	THEMIS
0	0.87168551	0.971	0.785	0	2	RORA
0	0.83114146	0.73	0.39	0	2	CADM1
0	0.769260223	0.862	0.53	0	2	RHOH
0	0.765469004	0.785	0.429	0	2	LINC02384
0	0.733319968	0.68	0.269	0	2	ALOX5AP
0	0.722064739	0.488	0.1	0	2	PZP
0	0.698684046	1	0.998	0	2	CD247
0	0.68825346	0.83	0.552	0	2	NPC1
0	0.682117295	0.999	0.965	0	2	PITPNC1
0	0.667323905	0.451	0.161	0	2	PTPRM
0	0.645631081	0.447	0.085	0	2	A2M
8.074E-305	0.672478505	0.812	0.539	1.8125E-300	2	SYTL2
1.1639E-292	0.645360448	0.848	0.6	2.6128E-288	2	TTC39C
1.17E-288	0.804087199	0.642	0.32	2.6264E-284	2	AC243829.2
4.0471E-287	0.782153635	0.842	0.632	9.0853E-283	2	CCNH
3.007E-282	0.625342227	0.835	0.575	6.7504E-278	2	GRAP2
2.4488E-236	0.620100102	0.827	0.587	5.4972E-232	2	ARHGAP26
1.3003E-227	0.744190317	0.349	0.125	2.9191E-223	2	PLCB1
0	1.310533923	0.928	0.491	0	3	SGCD

0	1.020350123	0.999	0.657	0	3	KLRC1
0	0.851996852	0.762	0.2	0	3	FCER1G
0	0.648403914	0.997	0.954	0	3	NEAT1
0	0.630385784	0.602	0.197	0	3	TNFRSF18
3.9635E-264	0.715987505	0.764	0.458	8.8977E-260	3	PHACTR2
2.1781E-247	0.451740756	0.249	0.06	4.8895E-243	3	PPP1R9A
3.4671E-219	0.631636229	0.258	0.071	7.7832E-215	3	PDE7B
3.5267E-212	0.440478178	0.472	0.208	7.9171E-208	3	ATP10A
2.4169E-200	0.431192333	0.997	0.973	5.4256E-196	3	LYST
1.1135E-194	0.559088923	0.859	0.642	2.4998E-190	3	PRSS23
1.2408E-181	0.442411026	0.844	0.563	2.7856E-177	3	SH2D1B
7.42E-173	0.428603354	0.416	0.187	1.6657E-168	3	RUNX2
2.3872E-161	0.492783518	0.925	0.782	5.359E-157	3	JAZF1
2.6042E-157	0.431817175	0.851	0.691	5.8462E-153	3	DSTN
3.6564E-155	0.421338365	0.957	0.778	8.2082E-151	3	CMC1
5.5578E-141	0.418562712	0.652	0.429	1.2477E-136	3	CTBP2
2.6811E-136	0.446506569	0.947	0.866	6.0188E-132	3	TGFBR3
1.1172E-128	0.428110548	0.825	0.653	2.5079E-124	3	PTPN12
1.472E-105	0.401081651	0.533	0.339	3.3046E-101	3	GCNT1
0	0.827156839	0.691	0.277	0	4	ALOX5AP
0	0.50978675	1	1	0	4	TMSB10
0	0.505924397	0.339	0.078	0	4	KLRB1
0	0.464811142	1	1	0	4	RPL10
0	0.398258407	1	1	0	4	B2M
3.401E-288	0.511086912	0.9	0.466	7.6348E-284	4	PDE3B
9.1136E-273	0.630630634	0.987	0.689	2.0459E-268	4	IL32
4.601E-252	0.603493267	0.978	0.913	1.0329E-247	4	S100A4
1.192E-231	0.486959366	0.988	0.947	2.6759E-227	4	TRBC1
7.811E-210	0.464345096	0.548	0.26	1.7535E-205	4	KIR3DL2
3.4798E-206	0.566188233	0.891	0.733	7.8118E-202	4	GIMAP7
1.1121E-183	0.573848436	0.896	0.727	2.4966E-179	4	CXCR4
1.722E-173	0.474289563	0.967	0.896	3.8657E-169	4	FGFBP2

6.3572E-171	0.414467856	0.682	0.365	1.4271E-166	4	THEMIS
8.7425E-157	0.441105681	0.845	0.545	1.9626E-152	4	CD3E
2.6418E-151	0.556820518	0.799	0.635	5.9306E-147	4	KLF2
1.2221E-147	0.389858395	0.975	0.946	2.7435E-143	4	PLAAT4
2.5963E-144	0.453285519	0.935	0.854	5.8284E-140	4	GZMA
1.6202E-128	0.392382934	0.973	0.945	3.6371E-124	4	CRIP1
3.3547E-128	0.497308121	0.578	0.337	7.5309E-124	4	AC243829.2
0	3.029375226	0.968	0.548	0	5	CCL4L2
0	2.844960024	0.684	0.143	0	5	XCL2
0	2.193224398	0.999	0.888	0	5	CCL4
0	1.61235024	0.89	0.513	0	5	CCL3
0	1.214854957	0.659	0.17	0	5	CRTAM
0	1.06665683	0.263	0.024	0	5	XCL1
0	0.667179927	0.356	0.081	0	5	TNF
0	0.626236863	0.417	0.097	0	5	TNFSF14
1.3252E-290	0.743594841	0.878	0.549	2.9749E-286	5	CD7
2.4389E-281	1.00450589	0.719	0.394	5.4752E-277	5	CD69
2.0792E-250	0.646480621	0.995	0.953	4.6676E-246	5	ACTB
6.942E-249	0.631416175	0.585	0.241	1.5584E-244	5	NR4A2
5.9312E-241	0.650883721	0.959	0.832	1.3315E-236	5	HOPX
9.5893E-234	0.655201208	0.986	0.699	2.1527E-229	5	IL32
1.4324E-214	0.857013473	0.406	0.14	3.2156E-210	5	CCL3L1
2.8042E-211	0.624440107	0.917	0.687	6.2952E-207	5	ZFP36L1
3.4561E-196	0.612784907	0.945	0.777	7.7586E-192	5	ARID5B
3.8052E-179	0.96594882	0.88	0.725	8.5423E-175	5	NFKBIA
8.9543E-158	0.648080621	0.765	0.542	2.0101E-153	5	REL
2.41479E-98	0.776890981	0.709	0.548	5.42096E-94	5	NFKB1
5.4381E-290	0.575283585	0.498	0.121	1.2208E-285	6	GOLGA8M
1.9303E-261	0.662424704	1	0.999	4.3333E-257	6	AOAH
8.1901E-258	0.75148193	0.615	0.204	1.8386E-253	6	TRIO
3.3723E-227	0.752992823	0.997	0.969	7.5706E-223	6	PITPNC1
8.0114E-202	0.872703574	0.704	0.318	1.7985E-197	6	CASK

1.7593E-190	0.72164282	0.54	0.196	3.9495E-186	6	AF165147.1
2.4689E-175	0.572732107	0.392	0.113	5.5424E-171	6	INPP4B
1.1031E-167	0.645517643	0.987	0.871	2.4764E-163	6	ATP8A1
1.3561E-156	0.685582677	0.971	0.823	3.0443E-152	6	SAMSN1
1.5835E-152	0.729851563	0.902	0.665	3.5548E-148	6	APBA2
5.3742E-130	0.688371762	0.865	0.613	1.2065E-125	6	AUTS2
1.0018E-125	0.605279093	0.989	0.963	2.249E-121	6	ZBTB20
2.5254E-117	0.5873872	0.504	0.227	5.6693E-113	6	GNAQ
1.2E-115	0.705949306	0.67	0.386	2.6938E-111	6	NCAM1
3.9851E-114	0.56593028	0.872	0.629	8.9461E-110	6	EML4
1.5795E-111	0.575385529	0.806	0.527	3.5459E-107	6	NEK7
2.80212E-92	0.670910885	0.479	0.238	6.29047E-88	6	KIAA0825
2.90978E-92	0.660444863	0.349	0.139	6.53215E-88	6	AC008014.1
8.58516E-86	0.58042817	0.788	0.541	1.92728E-81	6	TC2N
7.03918E-84	0.565026829	0.836	0.627	1.58022E-79	6	NIBAN1
0	1.577896856	0.643	0.031	0	7	ANK3
0	1.410985374	0.474	0.019	0	7	IL7R
0	1.406842182	0.479	0.015	0	7	GZMK
0	1.02295652	0.421	0.026	0	7	NELL2
0	0.756620032	0.368	0.016	0	7	UST
2.7348E-201	1.159241933	0.609	0.131	6.1394E-197	7	CAMK4
2.1727E-170	0.59019504	0.222	0.02	4.8775E-166	7	ITGA1
1.1738E-126	1.000943985	0.801	0.33	2.635E-122	7	CASK
8.9177E-117	0.645123497	0.6	0.189	2.0019E-112	7	TCF7
4.91153E-90	0.767013262	0.571	0.213	1.10259E-85	7	LINC00996
2.91223E-78	0.797652573	0.957	0.774	6.53766E-74	7	CD44
2.38376E-77	0.704321182	0.325	0.082	5.35131E-73	7	PCAT1
2.518E-77	0.747912224	0.904	0.625	5.65266E-73	7	FAM107B
1.11362E-71	0.849051079	0.863	0.548	2.49997E-67	7	TC2N
4.56386E-71	0.895987857	0.889	0.511	1.02454E-66	7	PDE3B
1.87555E-70	0.776603334	0.776	0.438	4.21041E-66	7	EPHA4
3.32628E-68	0.569427864	0.438	0.151	7.46716E-64	7	DRAIC

2.8722E-59	0.701142124	0.515	0.221	6.44781E-55	7	TRIO
4.71156E-44	0.637324937	0.84	0.635	1.0577E-39	7	NIBAN1
2.52299E-39	0.636832516	0.425	0.195	5.66386E-35	7	LINC02446
0	0.924257664	0.613	0.081	0	8	EGR2
5.8768E-276	3.033226258	1	0.58	1.3193E-271	8	CCL4L2
1.9879E-252	2.602523572	1	0.897	4.4626E-248	8	CCL4
4.8585E-234	1.29832846	0.881	0.261	1.0907E-229	8	NR4A2
1.3786E-181	1.141913364	0.634	0.155	3.0948E-177	8	CCL3L1
1.8309E-178	1.629564091	0.982	0.734	4.1101E-174	8	NFKBIA
1.3094E-175	1.065776397	0.854	0.266	2.9394E-171	8	FCER1G
5.6504E-149	1.705998447	0.946	0.541	1.2685E-144	8	CCL3
5.4781E-139	1.169622425	0.98	0.68	1.2298E-134	8	IGF2R
1.4555E-118	0.999451311	0.769	0.333	3.2674E-114	8	RELB
1.0566E-109	0.976833704	0.825	0.416	2.3719E-105	8	CD69
2.9434E-107	0.908149985	1	0.698	6.6077E-103	8	KLRC1
9.46227E-97	0.9771762	0.939	0.746	2.12419E-92	8	RAB8B
2.04556E-94	0.989724287	0.546	0.177	4.59207E-90	8	SNX9
9.24413E-93	0.922721863	0.946	0.704	2.07522E-88	8	ZFP36L1
8.07623E-86	1.03427604	0.656	0.283	1.81303E-81	8	GABPB1
3.27598E-85	0.939141499	0.906	0.667	7.35425E-81	8	RIN3
1.73338E-72	0.947002411	0.876	0.552	3.89126E-68	8	BNC2
3.88801E-69	1.206945278	0.813	0.558	8.72818E-65	8	NFKB1
1.62839E-66	0.876516787	0.951	0.821	3.65557E-62	8	IKZF3
0	3.316054372	1	0.02	0	9	LINGO2
9.38574E-91	0.751687377	0.76	0.27	2.10701E-86	9	FCER1G
1.78856E-51	1.011644296	0.858	0.547	4.01514E-47	9	SGCD
3.70154E-50	0.61124896	0.489	0.179	8.30958E-46	9	SNX9
6.33177E-50	0.736602696	0.927	0.701	1.42142E-45	9	KLRC1
1.77093E-46	0.750053855	0.765	0.44	3.97555E-42	9	GAS7
5.73679E-44	0.675582547	0.992	0.96	1.28785E-39	9	NEAT1
7.0493E-40	0.645501827	0.891	0.673	1.5825E-35	9	PTPN12
3.83519E-36	0.648633831	0.813	0.495	8.60962E-32	9	PHACTR2

1.6998E-35	0.521556497	1	0.976	3.81588E-31	9	LYST
5.55164E-33	0.560645626	0.944	0.799	1.24629E-28	9	JAZF1
4.92347E-32	0.494913771	0.961	0.881	1.10527E-27	9	PPP3CC
1.29653E-31	0.4975575	0.556	0.288	2.91058E-27	9	MTSS1
1.27605E-30	0.512866472	0.623	0.354	2.8646E-26	9	FAM3C
4.66105E-28	0.659262186	0.883	0.683	1.04636E-23	9	IGF2R
2.69569E-25	0.503084973	0.888	0.742	6.05156E-21	9	SLA
2.36193E-23	0.561380207	0.754	0.555	5.30229E-19	9	BNC2
8.91367E-21	0.518242684	0.589	0.373	2.00103E-16	9	GFOD1
1.31209E-18	0.494561112	0.813	0.67	2.94552E-14	9	PRSS23
5.78535E-15	0.502853394	0.472	0.293	1.29875E-10	9	BACH2
0	1.949327307	0.937	0.125	0	10	MX1
0	1.193829776	0.664	0.066	0	10	RSAD2
0	1.004214767	0.648	0.073	0	10	OAS1
0	0.928347843	0.513	0.024	0	10	IFIT1
1.8952E-243	1.553929484	0.83	0.178	4.2545E-239	10	MX2
1.6296E-211	1.330930754	0.736	0.147	3.6583E-207	10	HERC5
5.6047E-181	1.610442133	0.943	0.358	1.2582E-176	10	EPSTI1
2.2394E-178	2.132782496	0.975	0.502	5.0272E-174	10	ISG15
1.8086E-175	1.138991399	0.604	0.111	4.0602E-171	10	IFIT3
3.5117E-161	1.760178096	0.862	0.327	7.8835E-157	10	IFI6
1.5437E-155	1.451133938	0.909	0.371	3.4655E-151	10	EIF2AK2
1.1268E-149	1.435897391	0.921	0.42	2.5297E-145	10	XAF1
3.6592E-129	1.109003669	0.783	0.258	8.2146E-125	10	PARP9
2.9217E-128	1.357382299	0.953	0.589	6.5589E-124	10	LY6E
5.2248E-119	1.332441308	0.94	0.591	1.1729E-114	10	ISG20
1.5007E-114	1.053621279	0.755	0.27	3.3689E-110	10	STAT1
4.156E-111	0.935869836	0.802	0.3	9.3298E-107	10	IRF9
8.3373E-100	1.05335437	0.846	0.383	1.87163E-95	10	SAMD9L
7.0726E-80	0.978220542	0.915	0.58	1.58773E-75	10	PARP14
4.97532E-63	0.962994919	0.833	0.52	1.11691E-58	10	TRIM22
0	2.720090635	1	0.086	0	11	DLG2

2.62586E-27	0.600592511	0.908	0.676	5.8948E-23	11	APBA2
6.24247E-27	0.780863835	0.729	0.4	1.40137E-22	11	THEMIS
9.6768E-23	0.506203915	1	0.97	2.17234E-18	11	PITPNC1
1.32493E-22	0.453193567	1	0.999	2.97434E-18	11	AOAH
1.25038E-19	0.713471939	0.769	0.517	2.80698E-15	11	PDE3B
2.35443E-17	0.451108302	0.974	0.938	5.28546E-13	11	CCSER2
7.32925E-16	0.417456957	0.934	0.813	1.64534E-11	11	SLFN12L
1.63337E-14	0.432436724	0.83	0.641	3.66675E-10	11	EML4
6.82471E-14	0.42445913	0.799	0.611	1.53208E-09	11	GRAP2
7.62108E-14	0.376474453	0.603	0.364	1.71086E-09	11	EPSTI1
5.92613E-13	0.381947862	0.838	0.651	1.33036E-08	11	PRKCQ
6.7003E-13	0.439167238	0.764	0.553	1.50415E-08	11	TC2N
1.34599E-12	0.42704336	0.856	0.721	3.02161E-08	11	VTI1A
1.55393E-11	0.411425113	0.856	0.735	3.48842E-07	11	SOS1
1.60467E-11	0.369180382	0.611	0.409	3.60233E-07	11	JAKMIP2
2.1816E-11	0.370616953	0.991	0.964	4.89748E-07	11	ZBTB20
2.07435E-09	0.376644605	0.703	0.529	4.65671E-05	11	LINC00342
1.27412E-06	0.372043378	0.467	0.341	0.028602742	11	ST8SIA6
1.43575E-05	0.392325663	0.52	0.407	0.322312178	11	COMMD10
0	3.509622599	1	0.001	0	12	RRM2
0	3.251782343	1	0	0	12	MKI67
0	3.113151824	1	0.001	0	12	TOP2A
0	2.413698179	0.933	0.001	0	12	ASPM
9.7155E-249	2.44902331	1	0.013	2.181E-244	12	KNL1
6.6146E-172	2.436481371	1	0.019	1.4849E-167	12	CLSPN
2.1109E-115	3.178199913	1	0.029	4.7387E-111	12	CENPF
9.43523E-80	3.34249043	1	0.043	2.11812E-75	12	NUSAP1
8.86534E-59	4.576622443	1	0.06	1.99018E-54	12	STMN1
3.85093E-33	2.552047444	1	0.115	8.64495E-29	12	PCNA
5.29793E-27	2.744176963	1	0.145	1.18933E-22	12	EZH2
6.62427E-22	2.693926888	1	0.186	1.48708E-17	12	HIST1H1C
1.1111E-20	2.775764174	1	0.214	2.49431E-16	12	NSD2

1.62738E-19	2.726007299	1	0.235	3.65331E-15	12	ATAD2
1.06619E-16	4.28484422	1	0.307	2.3935E-12	12	TUBA1B
1.37282E-16	3.548338679	1	0.311	3.08184E-12	12	TUBB
2.58171E-15	2.517699102	1	0.35	5.79569E-11	12	SMC4
2.88611E-15	5.294662325	1	0.36	6.47903E-11	12	HIST1H4C
4.1699E-13	3.072757006	1	0.521	9.361E-09	12	HMGB2
2.6373E-12	2.822979344	1	0.681	5.92048E-08	12	HMGN2

P_val: raw p-value; avg_log2FC: log2-fold-change of the average expression between the two groups (here, cluster vs all cells); pct.1: fraction of cells where the feature is detected in the cluster tested; pct.2: fraction of cells where the feature is detected in the remaining clusters; p_val_adj: adjusted p-value, based on Bonferroni correction.

Table S4. Ordering of connected clusters in each lineage.

Lineage 1	Lineage 2	Lineage 3	Lineage 4	Lineage 5
**4**	4	4	4	4
2	2	2	2	2
**11**	11	11	11	11
**10**	10	10	10	10
**9**	9	0	6	5
**1**	1	7		
**8**	3			

Lineages are identified as a set of individual cells along a path/trajectory.
